# Modular microfluidics for life sciences

**DOI:** 10.1186/s12951-023-01846-x

**Published:** 2023-03-11

**Authors:** Jialin Wu, Hui Fang, Jun Zhang, Sheng Yan

**Affiliations:** 1 Institute for Advanced Study, Shenzhen University, Shenzhen, 518060 China; 2 Nanophotonics Research Center, Institute of Microscale Optoelectronics, Shenzhen University, Shenzhen, China; 3 Queensland Micro and Nanotechnology Centre, Griffith University, Brisbane, QLD 4111 Australia

**Keywords:** BioMEMS, Modular microfluidics, Biotechnology, Life Science, Microchannel connection

## Abstract

The advancement of microfluidics has enabled numerous discoveries and technologies in life sciences. However, due to the lack of industry standards and configurability, the design and fabrication of microfluidic devices require highly skilled technicians. The diversity of microfluidic devices discourages biologists and chemists from applying this technique in their laboratories. Modular microfluidics, which integrates the standardized microfluidic modules into a whole, complex platform, brings the capability of configurability to conventional microfluidics. The exciting features, including portability, on-site deployability, and high customization motivate us to review the state-of-the-art modular microfluidics and discuss future perspectives. In this review, we first introduce the working mechanisms of the basic microfluidic modules and evaluate their feasibility as modular microfluidic components. Next, we explain the connection approaches among these microfluidic modules, and summarize the advantages of modular microfluidics over integrated microfluidics in biological applications. Finally, we discuss the challenge and future perspectives of modular microfluidics.

## Introduction

Early in 1990, the term ‘micro total analysis systems’ (μTAS), now called lab-on-a-chip, was proposed to shrink the whole laboratory functionalities into a small microfluidic chip [1]. Although numerous microfluidics-enabled technologies have emerged, the original vision of operating chemical or biological laboratories at a microscale remains elusive.

Current microfluidic chips are largely still monolithic chips with a single purpose. They lack flexibility and cannot be adjusted in real-time based on different research works. After conducting a predetermined experiment, it has difficulties in performing the unplanned experimental protocols. Moreover, the monolithic chip with multiple functions requires professional skills in design and fabrication. This also discourages non-experienced biologists and chemists from using microfluidic chips. Therefore, a flexible, assemblable, standardized microfluidic platform would address the issues mentioned above, which can dramatically promote the development of microfluidic technology.

The idea of modular microfluidics is to deconstruct any microfluidic devices into their basic functional “units/modules” [2, 3]. These modules can be systematically designed and tested which reduce time and cost in prototyping. On the contrary, the integrated microfluidic chip that contains several modules but cannot flexibly configure additional functions is beyond the scope of this review [4]. Functioning modules can be assembled to fit the user’s experimental needs. For instance, connecting three individual organ chips (*i.e*., heart chips, liver chips, and fat chips) in series can achieve multi-organ microphysiological [5]. By extension of this concept, the human-on-a-chip modular microfluidic devices can be built by combining multiple organ chips, where each chip can mimic each part of the human body. Compared with an integrated multiorgan-on-a-chip plate, connecting individual organ chips is more flexible to the fit user’s experimental needs, reducing prototyping time and cost.

As an emerging field, modular microfluidics receives increasing interest from the microfluidics community in the field of life science, such as the advantage of controlling independent culture media. For example, tissues constructed from primary cells require specialized growth conditions to mature [6]. The modular method means that tissues on the chips can mature under specific conditions before the chips are connected, and then the chips are connected to research the interaction between different mature tissues.

**Fig. 1:**
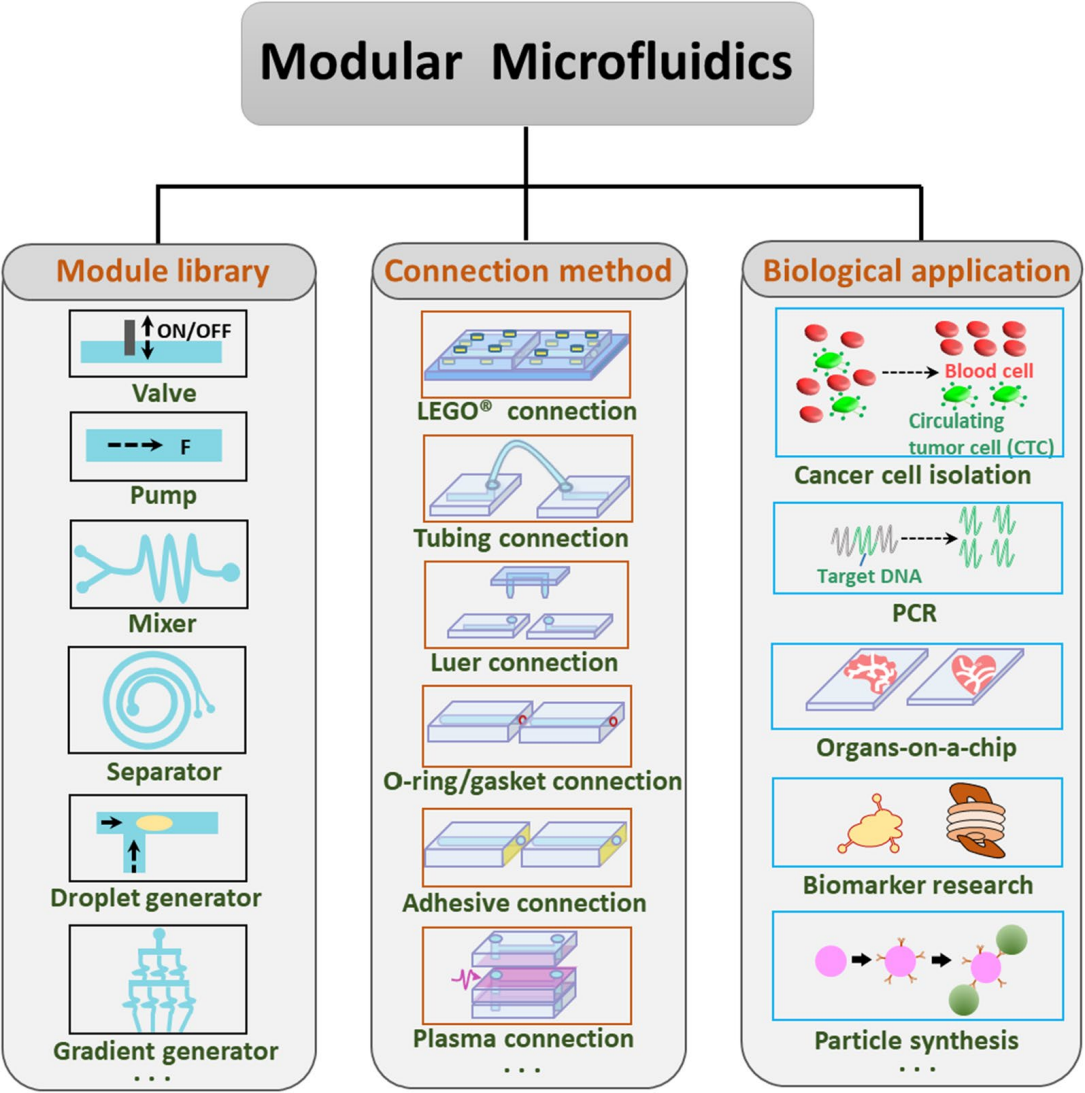
Outline of modular microfluidics. For different applications, the users can choose microfluidic modules and connection methods in the library to assemble the customized microfluidic system

Although a few recent literature review articles summarized the development of connection methods among modules [7, 8], the processing technologies, and applications of modular microfluidics, this review aims to focus on the channel connection between modules, summarize their merits and limitations in each application scenario, and summarize the benefits of modular microfluidics over integrated microfluidics in biological applications. This paper reviews the basic microfluidic modules, evaluates the feasibility of these basic modules as modular microfluidic components, evaluates the features of connection methods, summarizes the recent applications of modular microfluidics (Fig. 1), and proposes several promising trends for modular microfluidics.

## Microfluidic modules

A module is a complete functional component of the modular microfluidic system. In this section, we summarize the basic principles of main functional modules, including microvalve, micropump, micromixer, separator, droplet generator, gradient generator, trap and cell culture, and analyze their feasibility as the components of modular microfluidics.

### Microvalve

Microvalves regulate the fluid flow velocity and on–off in microfluidic systems. The ideal microvalve should be low cost, small size, easy integration, high flow control accuracy, no leakage and fast response. Microvalves control the magnitude and direction of fluid flow, which enables the precise and stable release of fluids in chemical or biomedical assays for point-of-care diagnostics [9] and drug delivery [10]. Microvalves can be categorized into active and passive types, according to the fluid flow control principle [11].

The active microvalves use external physical fields or chemical stimulation to activate mechanical and non-mechanical moving parts and control the flow fluid. In contrast, the operating state of the passive microvalves is determined by the fluids such as flow direction and fluid driving pressure [12].

#### Active microvalve

**Fig. 2:**
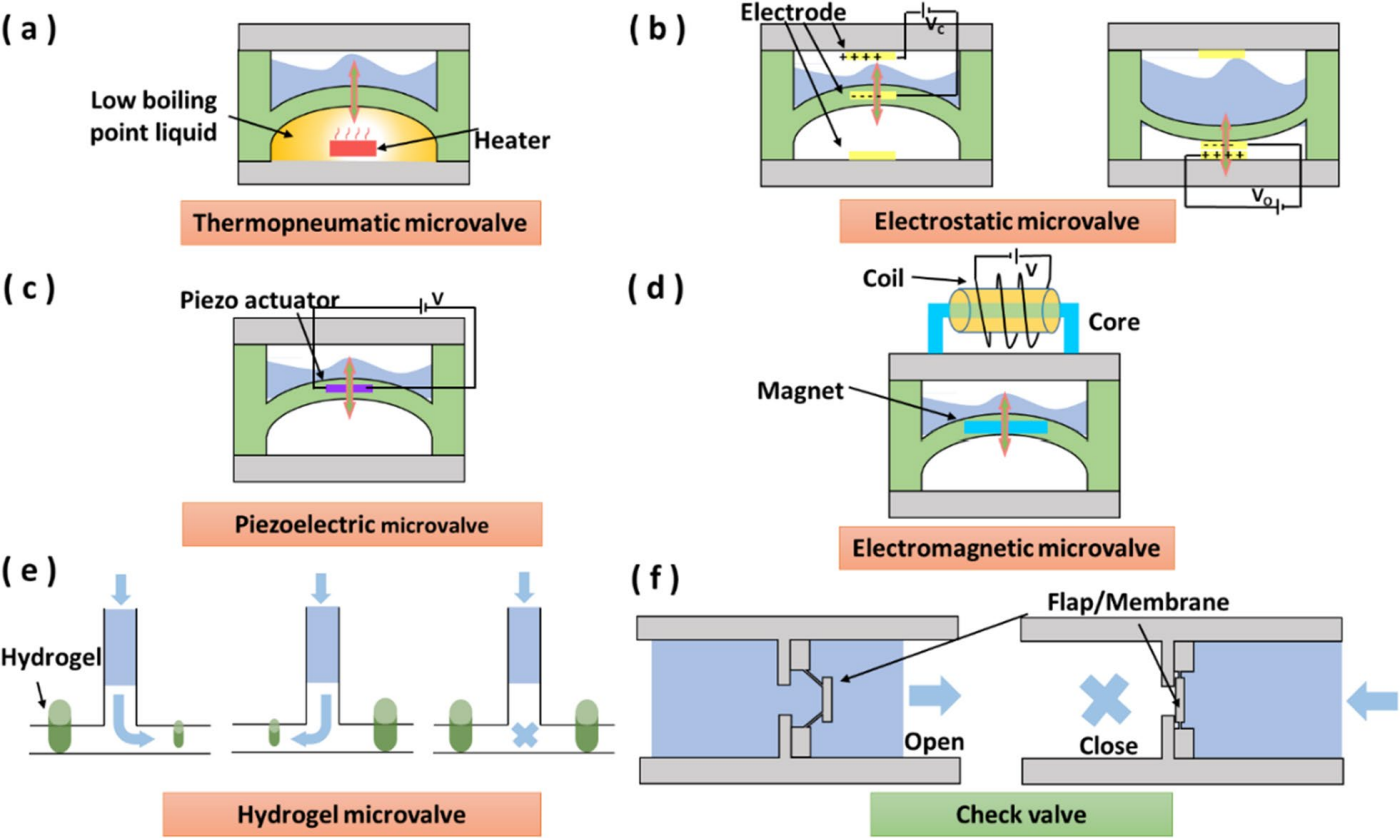
Schematic diagram of different microvalves. **a** Thermopneumatic microvalve. **b** Electrode microvalve. **c** Piezoelectric microvalve. **d** Electromagnetic microvalve. **e** Hydrogel microvalve. **f** Check valve

In active microvalves, thermopneumatic, electrostatic, piezoelectric and electromagnetic effects were used to deform mechanical membranes to control the fluid flow [12]. Thermopneumatic microvalves are operated through the volume thermal expansion of low-boiling point phase-change liquids [13] (Fig. 2a).

When the heater works, the phase-change liquid changes into the vapor state, which leads to the expansion of the chamber and the deformation of the elastic membrane. The advantages of thermopneumatic microvalves are their robustness and ability to generate large forces over a considerable distance. However, the limitation of thermopneumatic microvalves is their long response time, generally in the order of second even minute. For a quick response, an electrostatic force on the charged membrane can be used to control the fluid flow in the electrostatic microvalves [14] (Fig. 2b). The electrostatic microvalves respond quickly (response time is in the scale of several microseconds), and are suitable for instant fluid flow control with the fast response. Electrostatic microvalves are primarily used to control gas flow and are not eligible for liquids that are easily electrolysed [15–17].

Meanwhile, the piezoelectric crystal in piezoelectric microvalves produces internal tension or internal contraction force when an electrical voltage is applied, which results in valve plate to move and control liquid flow in the microchannel [18] (Fig. 2c). Piezoelectric microvalves apply to all types of fluids including gas and liquids. Due to its high-precision small displacement and fast response time [19], the piezoelectric microvalve is widely used in applications such as drug delivery [10, 20], ambulant blood pressure waveform monitoring [21] and micro-satellite [19]. However, piezoelectric microvalves are only suitable for small displacements. Electromagnetic microvalves can fill the demand for large displacement using an induced magnetic field to drive the mechanical membrane. When the electric current passes through the coil, an induced magnetic field will be generated around the coil. The magnetic field attracts the magnet to move upward, obstructing the liquid flow in the microchannel [22] (Fig. 2d). The electromagnetic microvalve is widely used in industrial devices because of its precise and real-time control.

Meanwhile, unlike the abovementioned active microvalves that directly apply the external physical forces on the diaphragm/membrane, an alternative method can employ “smart materials” such as stimuli-responsive hydrogels that can inflate by the chemical stimulation [23]. Under the stimulation of glucose concentration [24], pH value [24], temperature [25, 26], light [27] and other factors, the hydrogel volume can expand up to 1000 times [28]. Hydrogel microvalves can reversibly control fluid flow using the characteristic of reversible volume phase transitions (Fig. 2e).

#### Passive microvalves

Passive microvalves work based on the intrinsic structure in microchannels, and the fluid flow direction and driving pressure control the opening and closing states of the valves. Passive microvalves are categorized into check valves and capillary valves. Check valves can be applied to miniaturized microfluidic systems, while capillary valves are suitable for centrifugal microfluidic systems [29, 30]. However, centrifugal microfluidic systems usually need high-speed rotation to make the capillary valves obtain the driving pressure to open the valve, which is easy to break the connection of modules. Therefore, capillary valves are not suitable for modular microfluidic components.

In check valves, flap [31] or membrane [32] is ingeniously designed as the geometric structures in the microchannel to control the unidirectional flow of fluid. Because the fluid flow resistance is small in one direction and significant in the opposite direction (Fig. 2f), the check valve is also referred to as a “fluid diode”. Check valves can be made of various materials, including solid materials (e.g., Si [33]) and various polymers (e.g., parylene [34], PDMS [35] and SU-8 [36]). Specially, polymers are attractive materials for check valves because of their low cost and flexible manufacturing technology.

In summary, most active microvalves are highly sensitive and can accurately control the fluid. Thus, active microvalves are highly compatible with microfluid large-scale integration [37]. However, the active microvalves increase the complexity of the microvalve control system, bringing a challenge for the miniaturization of complex systems. Passive microvalves have shown excellent ability to work without external physical fields. Besides, compared with the active microvalves, the passive microvalves are lower in cost and power consumption and simpler in structure [38]. However, in passive microvalves, the fluid can only flow in one direction, which affects the pumping performance of a reciprocal displacement micropump [12].

In addition, all active microvalves here are suitable for modular microfluidic components because they can control the working state by adjusting physical fields. For passive microvalves, check valves, as the one-way valves, are also easily used in modular microfluidic systems, while capillary valves are not recommended to be used in modular microfluidics because they need to work in centrifugal microfluidic systems and are easy to break the connection of modules.

### Micropump

Micropump is a device that continuously delivers working fluid (liquid or gas) from the storage chamber to the designated location at a precise volume [39]. Therefore, the micropump is one of the core components of microfluidic systems and the power component to realize fluidic supply. According to the basic principle, micropumps can be categorized into mechanical micropumps and nonmechanical micropumps. The mechanical micropumps have the moving parts for liquid pumping, while the nonmechanical micropumps do not have [40].

#### Mechanical micropump

**Fig. 3:**
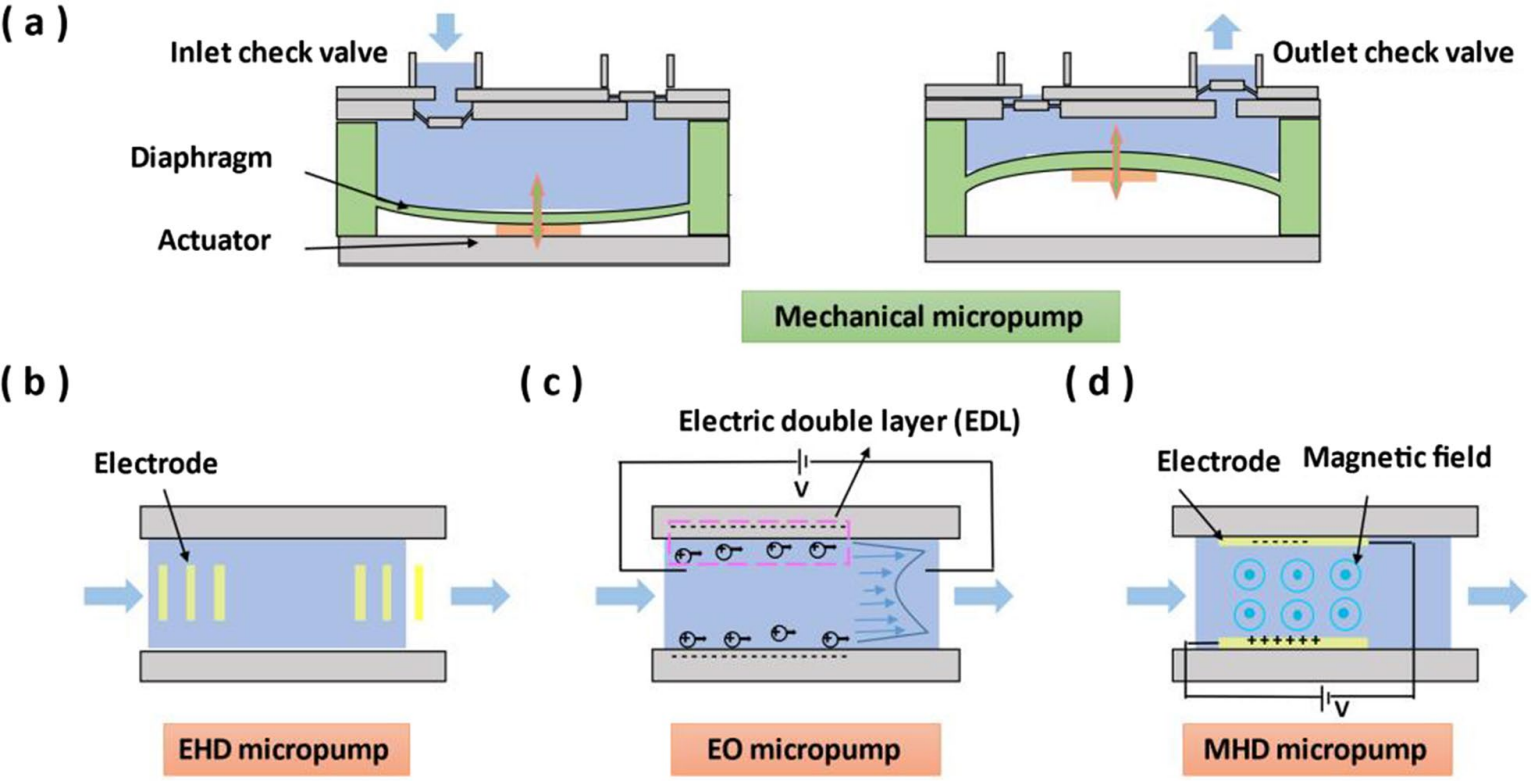
Schematic diagram of different micropumps. **a** Mechanical micropump. **b** EHD micropump. **c** EO micropump. **d** MHD micropump

Mechanical micropumps usually use moving mechanical parts, such as an oscillating diaphragm or a rotor, to directly apply pressure to drive the fluid [41]. Most reported micropumps are diaphragm/membrane type. The components of diaphragm micropumps generally include a pump chamber, a diaphragm, an actuator and two check valves [42] (Fig. 3a). Similar to abovementioned driving mechanisms of microvalves, the driving mechanisms of diaphragm micropump actuator include pneumatic [43], thermo-pneumatic [44, 45], electrostatic [46], piezoelectric [47] and electromagnetic mechanisms [48, 49]. The oscillation or displacement of diaphragm is driven by the actuator, which leads to the change of chamber volume and generates pressure difference to drive the fluid. The fluid enters the pump chamber from the inlet check valve, and then passes through the outlet check valve to complete a pumping process. However, the oscillation of the diaphragm can affect the smoothness of the output fluid [50]. Continuous rotary micropumps have the potential for extremely smooth fluid delivery. Its moving element is a microscale gear or vane that can be rotated electromagnetically [51, 52].

#### Nonmechanical micropump

Nonmechanical pumps rely on physical field to drive fluids, including electrohydrodynamic (EHD), magnetohydrodynamic (MHD) and electroosmotic (EO) methods. EHD micropumps utilize an electric field to pull ions due to the Coulomb force, which in turn pulls the bulk fluid through momentum transfer due to fluid viscosity [53–55] (Fig. 3b). EHD micropumps can delivery non-conducting dielectric fluids, while EO micropumps utilize the electrical field to pump conductive fluids. For EO micropumps, the fluid contacts with the charged wall of the microchannel to generate charges, which drives the liquid to move under an applied electric field [56] (Fig. 3c). Taking the negatively charged wall of the microchannel as an example, when the liquid contacts the negatively charged wall of the microchannel, the cations in the liquid gather on the wall of the microchannel to form a thin electric double layer (EDL) (0.1 nm-10 nm). Under the action of the applied electric field, the fluid is dragged by the cations due to the fluid viscosity to move from the anode to the cathode, completing the pumping action. The advantage of EHD and EO micropumps is that they are flexible to local fluid control by varying the applied electric field. However, the application of high voltage (~ 800 V) still brings safety and economic challenges [57].

The voltage requirement of MHD micropumps is less than that of EHD and EO micropumps, generally in the order of ~ 10 V [58]. For MHD micropumps, the passing conductive fluid is driven in the microchannel by the electromagnetically induced Lorentz force under the interaction of an applied current and an orthogonal magnetic field [59] (Fig. 3d). MHD micropumps are categorized into two types: direct current (DC)-operated and alternate current (AC)-operated [60]. DC-MHD micropumps use permanent magnets, which causes the fluid to be easily electrolyzed to generate bubbles and electrode degradation. AC-MHD micropumps can solve these problems very well. AC-MHD micropump uses the electromagnet, so an applied electric field and a magnetic field generated by an electromagnet synchronize high-frequency changes, causing chemical reactions to reverse quickly enough on electrodes. However, the energy consumption of AC-MHD micropumps is higher than that of DC-MHD micropumps [61].

In summary, most mechanical micropumps are mainly used to handle the working fluid with a high flow rate, generally in the order of several microliters to several milliliters per minute [40]. However, with the development of emerging biomedical technology, it is difficult for mechanical micropumps to accurately handle the working fluid with extremely low flow rate. Nonmechanical micropumps are applicable to the microscale channel because they convert other energy into kinetic energy of the fluid and can handle working fluids with extremely low flow rate, generally in the order of nanoliter per minute. However, nonmechanical micropumps are only applicable to liquids with specific properties, which limits their applications.

In addition, both mechanical micropumps and nonmechanical micropumps mentioned above can be modularized. Because they can flexibly pump fluid from upstream to downstream with the help of physical fields, without affecting the breakage of the module connection.

### Micromixer

Rapid mixing of fluids is essential in microfluidic applications such as drug delivery, sequencing, amplification, and biochemical reactions. However, the fluid in the microchannel is usually laminar, and the Reynolds number (Re) is less than 1, which is very stable. The traditional mixing of fluids mainly depends on diffusion, which is very inefficient. Fluids with high Re can form chaotic flow, which shortens the mixing length and improves the mixing efficiency [62]. Mixing mainly relies on molecular diffusion and chaotic advection. Micromixers can be categorized into passive micromixers and active micromixers.

#### Passive micromixer

**Fig. 4:**
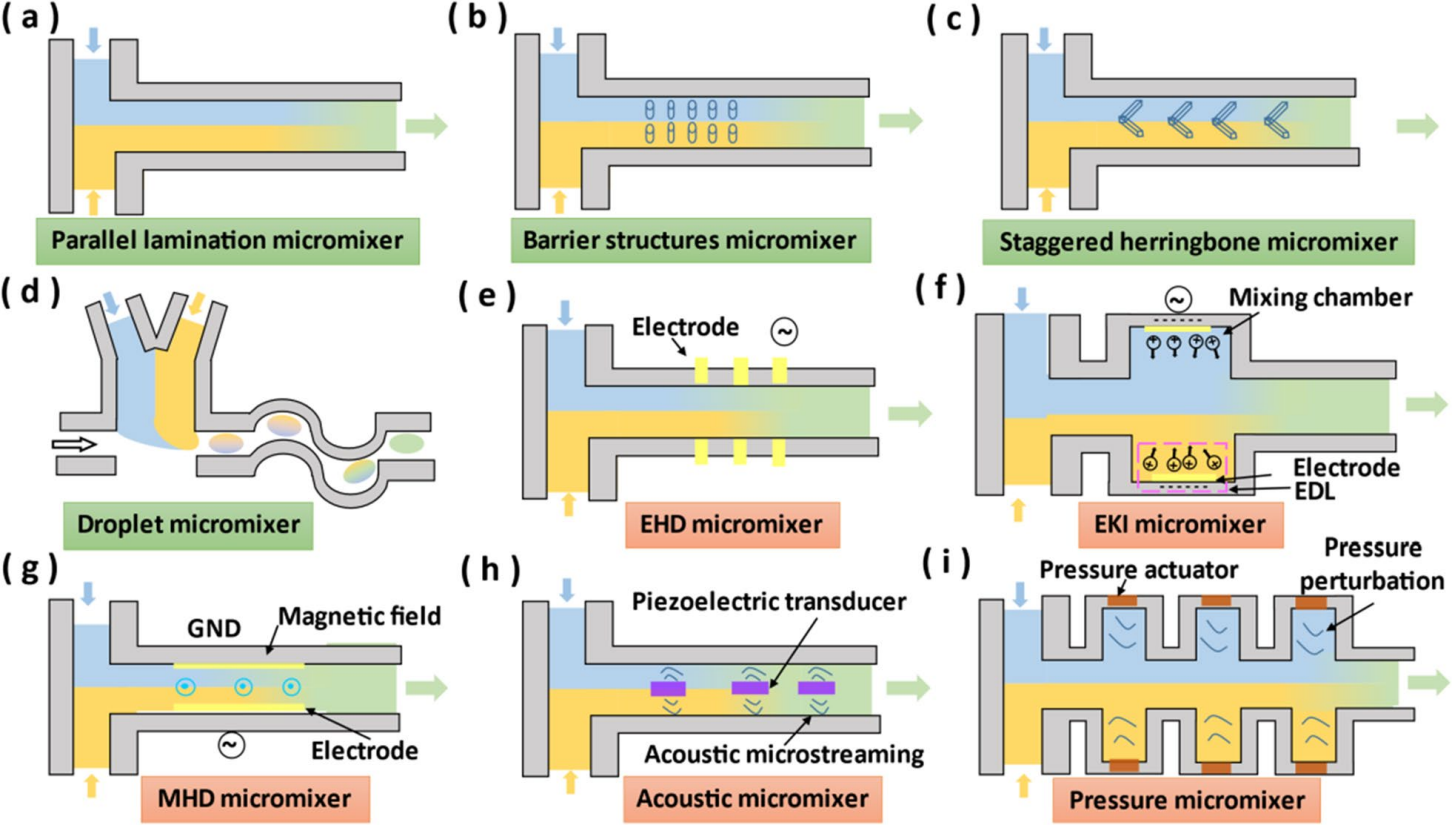
Schematic diagram of different micromixers. **a** Parallel lamination micromixer. **b** Barrier structures micromixer. **c** Staggered herringbone micromixer. **d** Droplet micromixer. **e** EHD micromixer. **f** EKI micromixer. **g** MHD micromixer. **h** Acoustic micromixer. (i) Pressure micromixer

According to the basic principle, passive micromixers can be categorized into three types, including lamination, chaotic advection and droplet [63]. Lamination micromixers can be designed in parallel and serial types. Parallel lamination micromixer is the simplest micromixer (Fig. 4a). Its design principle is that sub-streams enter long mixing microchannels simultaneously from several inlets respectively, enhancing the mixing effect by increasing the contact surface area between sub-streams [64–67]. However, parallel lamination micromixers simply rely on the diffusion of molecules between different concentrations of fluid layers in long mixing microchannels, which is difficult to achieve fast and efficient mixing [68]. Therefore, it is necessary to design microchannels to allow more surface area contact between different fluid layers. Serial lamination micromixers are referred to as split-and-recombine (SAR) micromixers. Specifically, within SAR micromixers, a fluid flowing horizontally is split vertically into sub-streams, and these sub-streams are recombined horizontally at the next confluence point of the microchannel. Repeat this process multiple times to achieve an exponential increase in contact surface area of fluid and a decrease in mixing length [69].

Advection generally occurs parallel to the direction of main fluid, so advection limits the lateral mixing of the fluid. To increase the mixing efficiency of the advection, the fluid needs to be stretched and folded across the cross-section of the microchannel, resulting in advection in other directions, also known as chaotic advection. The geometry or topography of the microchannel and the Reynolds number are the key parameter to modulate the chaotic advection [63]. For high Re fluids (Re > 100), the easiest way is to insert barrier structures on the mixing microchannels [70, 71] (Fig. 4b). For fluids with intermediate Re (10 < Re < 100), Tesla structure [72, 73] and three-dimensional serpentine structure [74, 75] are suitable. For low Re (Re < 10), the previous method has difficulty in producing chaotic advection. An effective solution is to form rips or grooves on the microchannel walls, such as slanted ridges mixer and staggered herringbone mixer [76] (Fig. 4c). Another way to improve mixing efficiency is to encapsulate the fluid to be mixed into the droplet emulsions (Fig. 4d). The fluid to be mixed is confined within the tiny geometric size of the droplet. The process of the droplet passing through the serpentine microchannel will also induce chaotic advection inside the droplet, leading to the full contact of the internal fluid and accelerated mixing [77, 78].

#### Active micromixer

Active micromixers utilize the disturbance generated by the applied physical field for the mixing effect. For active micromixers, applied physical fields can be categorized into EHD, electrokinetic instability (EKI), MHD, acoustic and pressure field [63].

The basic principle of EHD micromixers is to apply current to the electrodes placed in the microchannel, and electrically charged fluids generate secondary flow by electric force to improve the mixing efficiency (Fig. 4e). The applied current can be DC or AC, and the direction of the electrodes can be perpendicular [79] or parallel [80] to the direction of the main fluid. EHD micromixers are suitable for leaky dielectrics fluids, while EKI micromixers can utilize the alternating electrical field to mix conductive fluids [81, 82]. To mix conductive fluids, the conductivity gradient is formed in EKI micromixers by the uneven interface charges and drives the fluid to move (Fig. 4f). Under the AC field, the fluid becomes instable and mixes in the chambers [81]. However, both EHD and EKI micromixers need high electrical potentials, generally in the order of ~ 200 V, which may cause damage to biological samples. MHD micromixers usually require only a low electrical potential, generally in the order of ~ 5 V. For MHD micromixers, electrolyte solution is moved by the Lorentz force in mutually orthogonal electric and magnetic fields [83–85] (Fig. 4g). By changing the direction of current or magnetic field, the fluid is rolled and folded, which can induce secondary flows for stirring and mixing.

EHD, EKI and MHD micromixers are effective under the specific working fluids, while acoustic and pressure field-based micromixers are suitable for any working fluids. Acoustic micromixers rely on piezoelectric transducers placed in microchannels to produce acoustic streaming perpendicular to the direction of the mainstream [86] or utilize an audio speaker to axially oscillate the inlet tube of the microfluidic platform [87], which are used to stir and mix fluids (Fig. 4h). In addition, acoustic micromixers can also use bubbles in the liquid to enhance the mixing effect [88, 89]. Specifically, bubbles in liquid media are excited and vibrate at a specific resonant frequency of the sound field. This vibration causes acoustic microstreaming around the bubbles, which produces the strong liquid circulation flow and greatly enhances mixing efficiency. However, the temperature will rise with the energy transfer of the high-frequency sound field, which may damage the biological fluid. To overcome the problem of temperature rise, pressure micromixers are developed to realize mixing by disturbing the fluid through the pressure field without the temperature rise. The disturbance of pressure field can be achieved by alternating on–off input mechanism of each sub-fluid [90] or by generating of fluidic pulsing velocity in multiple-side channels [91, 92] (Fig. 4i).

In summary, passive micromixers use various obstacles to generate vortex for liquid mixing. Passive micromixers are characterized by simple structure, stable operation, easy integration and low in cost. However, for fluids with very low Re (Re < 1), the mixing performance of passive micromixers are ineffective. Active micromixers can improve the mixing performance by applying an external force to the fluid with very low Re to accelerate the diffusion process. However, active micromixers may damage biological samples with the high-frequency mixing and high-energy radiation.

In addition, both passive micromixers and active micromixers mentioned above can be modularized. Because they can effectively mix the upstream fluids and obtain the uniformly mixed fluid in the downstream of modules, with the help of geometry or physical field, without breaking the module connection.

### Separator

Highly uniform particle groups, such as beads, cells, vesicles and drops are of great significance in the fields of biomedicine, medicine research and chemical analysis. The role of separator is to separate these target particles from mixtures with different characteristics, so the separator is one of the most important components of a microfluidic system. Separators can also be categorized into active and passive types [93]. Active separators use external physical fields to separate particles, while passive separators rely on the interaction between particles, the geometry of the microchannel and the flow field to achieve separation.

#### Active separator

**Fig. 5:**
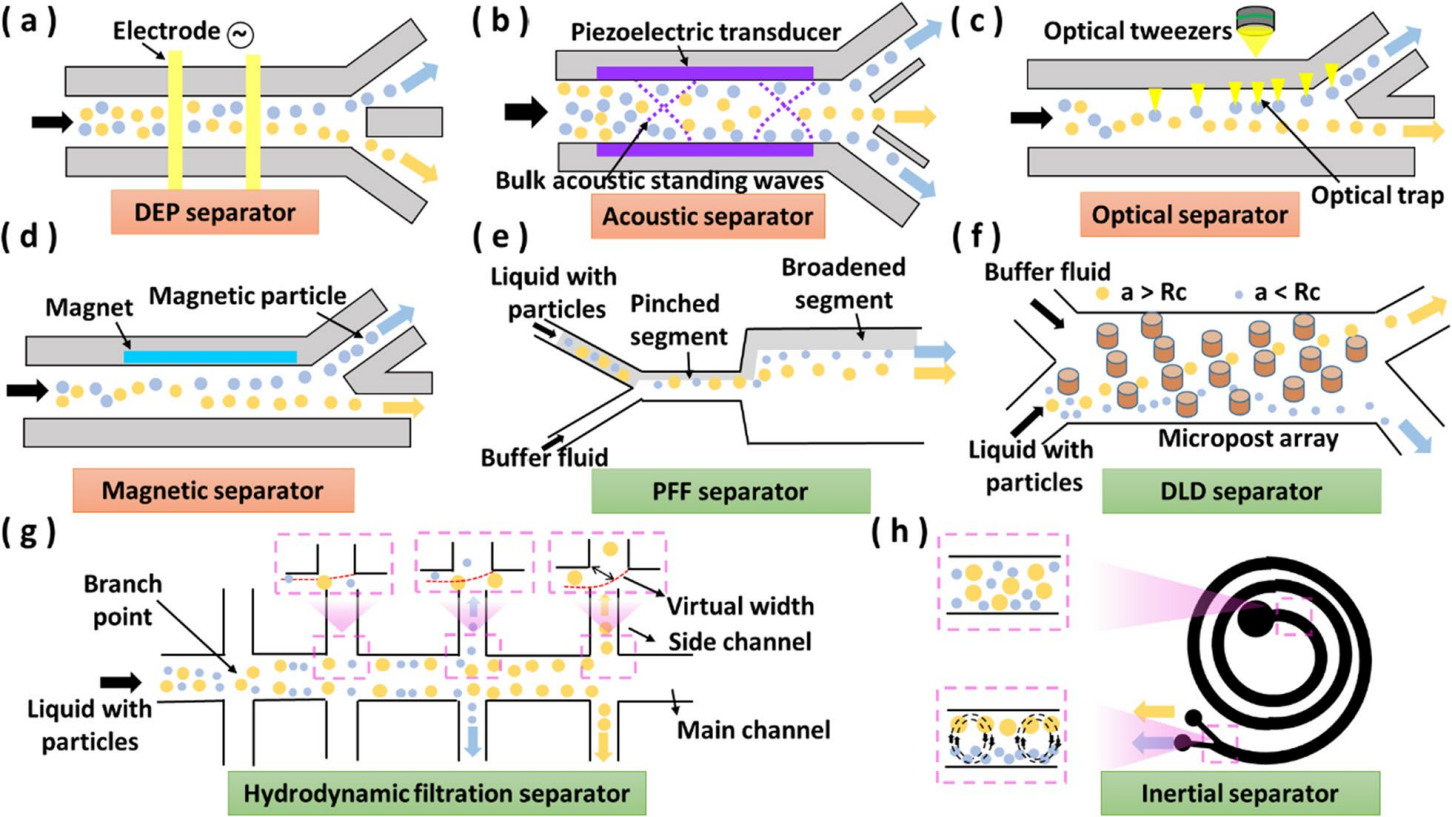
Schematic diagram of different separators. **a** DEP separator. **b** Acoustic separator. **c** Optical separator. **d** Magnetic separator. **e** PFF separator. **f** DLD separator. **g** Hydrodynamic filtration separator. **h** Inertial separator

Active separation includes dielectrophoretic (DEP), magnetophoretic, acoustophoretic and optofluidic methods. DEP separator refers to the displacement phenomenon of dielectric particles in a non-uniform electric field because of the polarization effect by the DEP force [94]. The DEP force depends on the size and shape of particles, the difference in the permittivity of particles and the surrounding medium, and the frequency of the electric field [95]. Thus, DEP separators can rely on these factors to separate particles with different properties in the medium (Fig. 5a). In a non-uniform electric field, when the particle is more polarized than the surrounding medium, the particle will move to the region of the strong electric field, which is called positive dielectrophoresis (pDEP). Conversely, the particle moves towards the region of the weak electric field, which is called negative dielectrophoresis (nDEP). Non-uniform electric fields can be created by using electrodes of various shapes [96], or by applying voltages along microchannels containing obstacles, such as ridges [97] or posts [98]. However, in DEP separators, electrodes are usually in contact with the medium, in which higher current tends to degrade the electrodes.

For acoustic, optical and magnetic separators, their actuators do not contact with the medium, overcoming the problem of electrode damage. The principle of acoustic separation is that in an acoustic standing wave of a half wave length, the suspended particles in the microchannel are subjected to acoustic radiation forces and move to the pressure node (the center of the microchannel) or antinode (microchannel wall) [99, 100] (Fig. 5b). The direction of movement depends on the acoustic contrast factor $\phi$ (Eq. 1).

1 $$\phi =\frac{5{\rho }_{c}-2{\rho }_{w}}{2{\rho }_{c}+{\rho }_{w}}-\frac{{\beta }_{c}}{{\beta }_{w}}$$

The densities of the medium and particles are denoted ${\rho }_{w}$ and ${\rho }_{c}$ respectively and the corresponding compressibilities ${\beta }_{w}$ and ${\beta }_{c}$ respectively. When $\phi$-factor is positive, particles move to the pressure node, and when $\phi$-factor is negative, particles move to the antinode. Therefore, when particles enter the microchannel, they will move toward either the pressure node or the antinode, which depends on the density and compressibility of the particles and the medium respectively.

Meanwhile, the optical separation is another separation method with no damage to the electrode and no requirements for the solution. When a beam of Gaussian light hits a particle, the light scatters and refracts and its direction changes. The difference between the initial and final momentum of the light is transferred to the particle. If the particle is off-center from the beam, the particle is driven by a net resultant force directed towards the center of the beam and trapped at the beam's strongest point, also known as optical tweezers [101, 102] (Fig. 5c). Optical separators can separate various target particles based on the difference in particle size or refractive index [103].

Compared with DEP, acoustic and optical separators, magnetic separation is the only technique that can separate particles with magnetic properties. Magnetic separators rely on magnetophoresis, which causes magnetic particles or magnetically labeled cells to move in the direction that they are attracted or repelled by the magnet when they subjected to a non-uniform magnetic field [104, 105] (Fig. 5d). The force (*F*_*m*_) acting on a magnetic particle within a magnetic field depends on the volume of the particle (*V*_*p*_), the difference between magnetic susceptibilities (*∆χ*) of the particle (*χ*_*p*_) and the surrounding buffer medium (*χ*_*m*_), as well as the strength (*B*) and gradient (*∇*) of the magnetic field [106]:

2 $${F}_{m}= \frac{{V}_{p}\cdot \Delta \chi }{ {\mu }_{o}}\left(B\cdot \nabla \right)B$$

However, the magnetic bead labeling technique takes long incubation and is manually intensive. To avoid the labeling process, magnetic separators can separate non-magnetic particles or cells of different sizes by modifying the surrounding buffer medium (e.g., adding water-based ferrofluids) to increase the magnetic susceptibilities [107].

#### Passive separator

Passive separators work based on the intrinsic structure in microchannels and the relative velocity of each fluid. Passive separation includes pinched flow fractionation (PFF), deterministic lateral displacement (DLD), hydrodynamic filtration and inertia separation. Among them, the flow rate of PFF, DLD and hydrodynamic filtration is relatively slow, mainly within a few microliters per minute. For PFF, the two inlets are liquid with particles and buffer without particles, respectively [108]. When the liquids enter the pinched segment, the liquid flow with particles is focused on one sidewall in the pinched segment (Fig. 5e). When the liquids are at the boundary between pinched segment and broadened segment, larger particles experience a force towards the center of the microchannel, while smaller particles experience a force towards the sidewall. Thus, particles of different sizes can be separated in the broadened direction perpendicular to the flow segment.

Another way to classify particles by size is DLD [109]. Specifically, when particles of different sizes pass through each layer of a periodic array of micropost, particles larger than the critical radius (a > Rc) and particles smaller than the critical radius (a < Rc) move in different directions [110] (Fig. 5f). This process is repeated and accumulated continuously, and the particles are categorized into two paths after passing through the array.

Meanwhile, hydrodynamic filtration is a separation method that requires neither a particle-free buffer nor control of inlet flow [111, 112]. When the relative flow rate of side channel at a branch point is sufficiently low and the diameter of the particle is larger than a specific value (a virtual width), the particle will not enter the side channel, even if the particle diameter is smaller than that of the side channel (Fig. 5g). The virtual width of the side channel increases with the increase of the flow rate of the side channel. If the diameter of the particle is smaller than the virtual width of the side channel, it can enter the side channel. Therefore, controlling the flow rates of side channels can effectively separate particles of different sizes.

The flow rate of inertial separators is relatively high, mainly form tens of microliters per minute to hundreds of microliters per minute. In the straight channel, inertial separation relies on inertial lift forces, so that particles of different sizes are fixed at different lateral equilibrium positions after passing through special geometric microchannels [113–116]. The inertial force comes from the boundary effect of the fluid flow adjacent to the wall of the microfluidic channel, and the magnitude of the force is related to the size of the particle. In a curved channel, the secondary flow is induced by a radial pressure gradient due to the mismatch of fluid momentum in the central and near-wall regions within the curvature, forming two counter-rotating streams, known as Dean vortices (Fig. 5h). This secondary flow has several benefits in inner focusing, including changing the inertial equilibrium positions, promising complete particle separation and reducing the channel length [117].

In summary, the efficiency and accuracy of active separators are excellent. However, the applied physical field will increase the complexity of microfluidic chip fabrication. In contrast, passive separators are work based on the intrinsic structure and the flow field in microfluidic chip, which makes microfluidic chips easier to fabricated and have better portability. Nevertheless, the accuracy of passive separators is less than that of active separators.

In addition, both active separators and passive separators mentioned above can be modularized. Because they can effectively separate mixed particles which are from upstream, and obtain particles of different features in downstream, without breaking the module connection.

### Droplet generator

Droplet in microfluidics refers to a suspended and deformable emulsion microdroplet formed by a liquid (dispersed phase) surrounded by a second immiscible liquid (continuous phase). The droplet interface acts as a membrane that confines its contents, helping to precisely control the chemical reactions inside, and the droplet can act as a container for transporting materials and chemical reactions, which can prevent sample dispersion and channel wall contamination [118]. Compared with the traditional macroscopic operating conditions of chemical reactions, droplets in microfluidics can rapidly analyze extremely small quantities of reactants in a portable, automated and inexpensive form [119]. In addition, the properties of droplets depend on the size of the droplet and the homogeneity of the internal sample, so the generation of droplets with controllable size and high dimensional accuracy is very important for the accuracy and reproducibility of microfluidic experiments.

Specifically, viscous force and interfacial tension are two important forces during the extension, deformation and breakup of droplets. The capillary number (Ca) is the ratio of viscous force to interfacial tension, and its formula is as follows:

3 $$Ca=\frac{\mu U}{\gamma }$$

where $\mu$ is the fluid dynamic viscosity, $U$ is the fluid velocity, and $\gamma$ is the interfacial tension between the two fluids [120]. Ca determines the droplet generation mode, where increasing Ca will lead to the transition of the droplet from squeezing to dripping, while further increasing Ca will lead to the transition to the jetting state. Droplet generators can generally be categorized into passive and active [121]. The passive droplet generators rely on hydrodynamic pressure of the flow and microscopic geometry to control the local flow field, thereby controlling the deformation and breakup of each droplet. The active droplet generators are that external physical field is locally added to the droplet formation area to achieve droplets generation.

#### Passive droplet generator

**Fig. 6:**
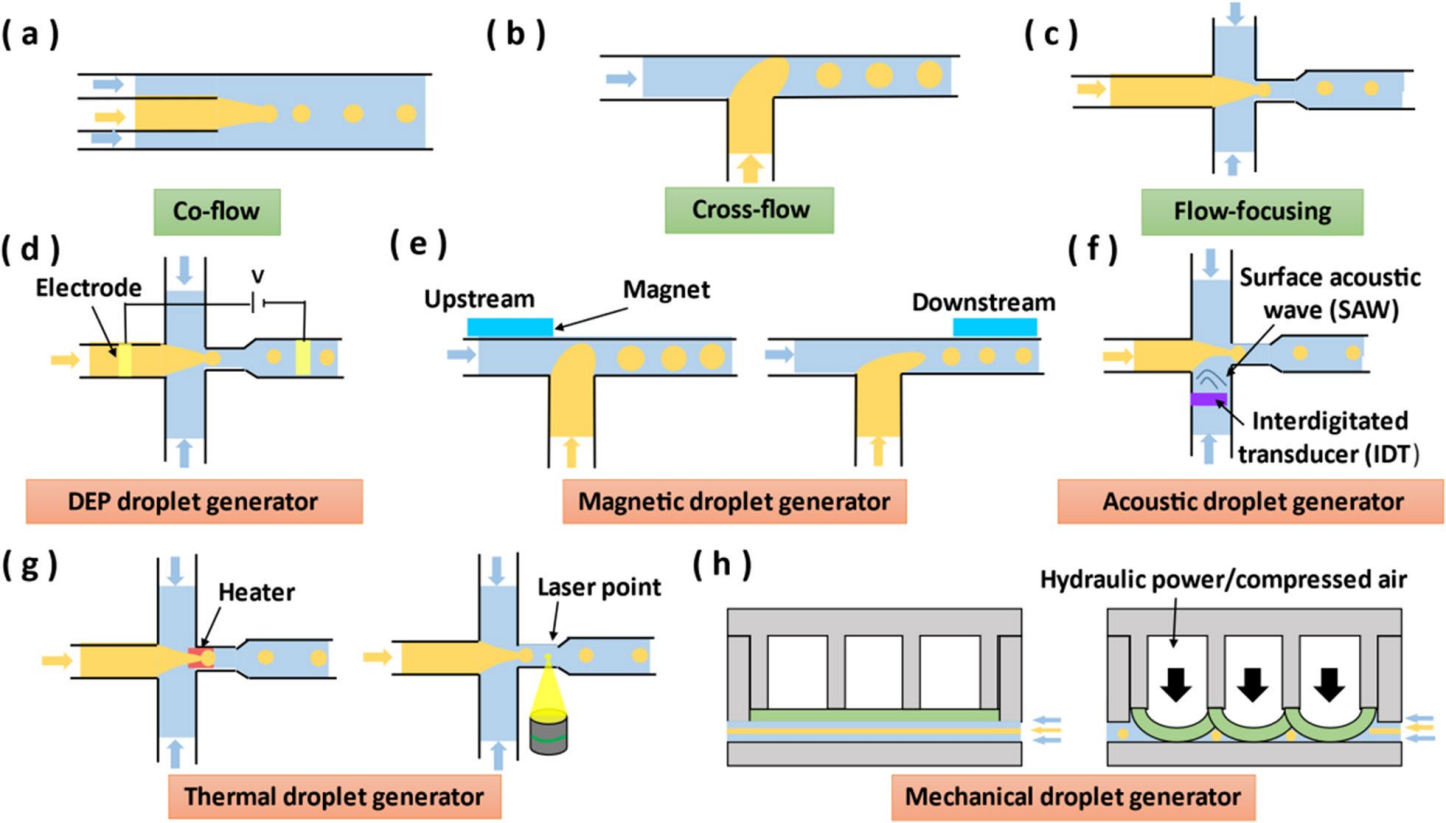
Schematic diagram of different droplet generators. **a** Co-flow droplet generator. **b** Cross-flow droplet generator. **c** Flow-focusing droplet generator. **d** Electrical droplet generator. **e** Magnetic droplet generator. **f** Piezoelectric droplet generator. **g** Thermal droplet generator. **h** Mechanical droplet generator

Passive droplet generators include co-flow, cross-flow and flow-focusing [122]. The simplest geometry in a droplet generator is a set of concentric channels in which the dispersed phase liquid is driven into the inner channel or capillary and from the end of the capillary into the continuous phase flowing stream, also known as co-flow [123] (Fig. 6a). The flow rates of the continuous and dispersed phases can determine the droplet size and droplet formation mechanism. Cross-flow is one of the most commonly used microfluidic droplet generation methods, and the typical structure is T-junction [124] (Fig. 6b). As the disperse-phase fluid blocks the continuous-phase fluid flow, a shear gradient is created, causing the disperse-phase fluid to elongate and eventually break into droplets. The flow rates and viscosities of the two phases, and the width of the microchannels affect the size of droplets [125, 126].

Compared to co-flow and cross-flow, flow-focusing can generate smaller droplets because a small orifice can affect the breakup process of the passing fluid. In flow-focusing geometry, the outer fluid flows coaxially with the inner fluid through a small orifice, then the outer fluid forces the inner fluid breaks inside or downstream of the orifice [127] (Fig. 6c). Likewise, the flow rate and fluid viscosity of the two phases can affect the droplet size formed.

#### Active droplet generator

Active droplet generation can be categorized into DEP, magnetic, acoustic, thermal and mechanical methods according to the applied physical field. For DEP droplet generators, the application of the electric field induces the accumulation and migration of charges at the droplet interface [128, 129] (Fig. 6d). When a high voltage is applied to the electrodes placed in the main channel, the charges accumulated on the surface of the droplet are subjected to electric force, which attracts the droplet to areas of higher electric field intensity and affects the process of droplet breakup. Without changing the flow rate of the two-phase fluid, the droplet size decreases with the increase of the applied electric field. In DEP droplet generators, the fluid can be electrically neutral, but the dielectric permittivity of droplet should be higher than that of the surrounding fluid. In addition, for magnetic fluids, such as ferrofluid, the use of an external magnetic field to control droplet formation is a good approach. Ferrofluids are a class of nanofluids containing magnetic nanoparticles that are magnetized by applying a magnetic field and demagnetized when the magnetic field is withdrawn. As shown in Fig. 6e, when a magnetic field is applied upstream or downstream of the T-junction, the magnetic force pulls back or pushes the emerging ferrofluid droplet, delaying or accelerating the droplet formation, which results in the formation of larger or smaller droplets [130]. The size of the droplet is affected by the properties of the magnetic field, such as magnetic field strength, location, orientation or magnetic flux density [131].

Acoustic, thermal and mechanical droplet generator don’t have requirements for the working fluid. Acoustic droplet generator can utilize interdigitated transducer (IDT) to generate the surface acoustic wave (SAW) in the junction. Due to the influence of the applying SAW, the droplet will produce asymmetry in the neck before the pinch-off, which reduces the liquid length of the generated droplet, resulting in the reduction of the droplet size and the increase of the generation speed [132–134] (Fig. 6f). In addition, since the viscosity and interfacial tension of fluids vary with temperature, thermal approaches can also be used to control droplet production (Fig. 6g). There are two methods of heating. One is to place a micro heater at the junction, and the diameter of the droplet is proportional to the ratio of interfacial tension to viscosity [135]. The other is to flexibly adjust the laser position, the advancing droplet interface was blocked at the laser spot and the volume in the water tip increases until the viscous stresses break it off. Therefore, the laser delays the formation time of the droplet, increasing the volume of the droplet [136].

Compared with the on-chip integration (e.g., electrode, piezoelectric transducer and microheater), the off-chip mechanical vibrator is simpler in assemble [137].In the mechanical droplet generator, the external mechanical vibrator (e.g., elastic valves or membranes) deflected by hydraulic power or compressed air, such that the continuous phase and the dispersed-phase liquids are “chopped” to generate independent and uniform droplets [138, 139] (Fig. 6h). The droplet generation process can be precisely controlled by adjusting the amplitude and the frequency of the mechanical vibrator [140].

In summary, passive droplet generators are simple in structure, easy to fabricate and portable. However, passive droplet generators have hysteresis in regulating droplet properties, generally in the order of seconds or even minutes. Compared with passive droplet generators, active droplet generators have higher flexibility and shorter system response time in controlling droplet size and production speed [137]. However, active droplet generation also increases the complexity of microfluidic chip.

In addition, both passive droplet generators and active droplet generators mentioned above can be modularized. Because they can be used as individual functional modules to effectively generate droplets inside themselves, without affecting the operation of other modules and damaging the module connection.

### Gradient generator

In vivo, concentration gradients of biomolecules regulate cellular behavior, such as cell growth and differentiation. In addition, concentration gradients of chemicals play important roles in many biological phenomena, including inflammation, wound healing, and cancer metastasis [141]. For chemical analysis, gradient generation can also be applied to drug development and chemical synthesis. Traditional concentration gradient experiments, such as in multi-well plates, require relatively large volumes of reagents, and the control of the concentration gradient is not precise and constant, and the gradient cannot be adjusted quickly. The gradient generators in the microfluidic system only require a small number of reagents, which is very beneficial for experiments using expensive or scarce reagents. Furthermore, the gradient generators in the microfluidics can maintain long-term static stability and dynamic fast response [142–145].

The gradient generators mainly include convection-based, diffusion-based and droplet generation-based. For convection-based gradient generators, in laminar flow, miscible fluids flow side by side and mix by diffusion. In diffusion-based gradient generators, membranes or hydrogels are often used to provide high resistance of the microchannel, so the solute in fluids can only slowly generate gradient through diffusion. Besides, droplet-based gradient generators are used to generated gradients in the droplet array, where the concentration of the dispersed phase can be adjusted by the inlet flow rate.

#### Convection-based gradient generator

**Fig. 7:**
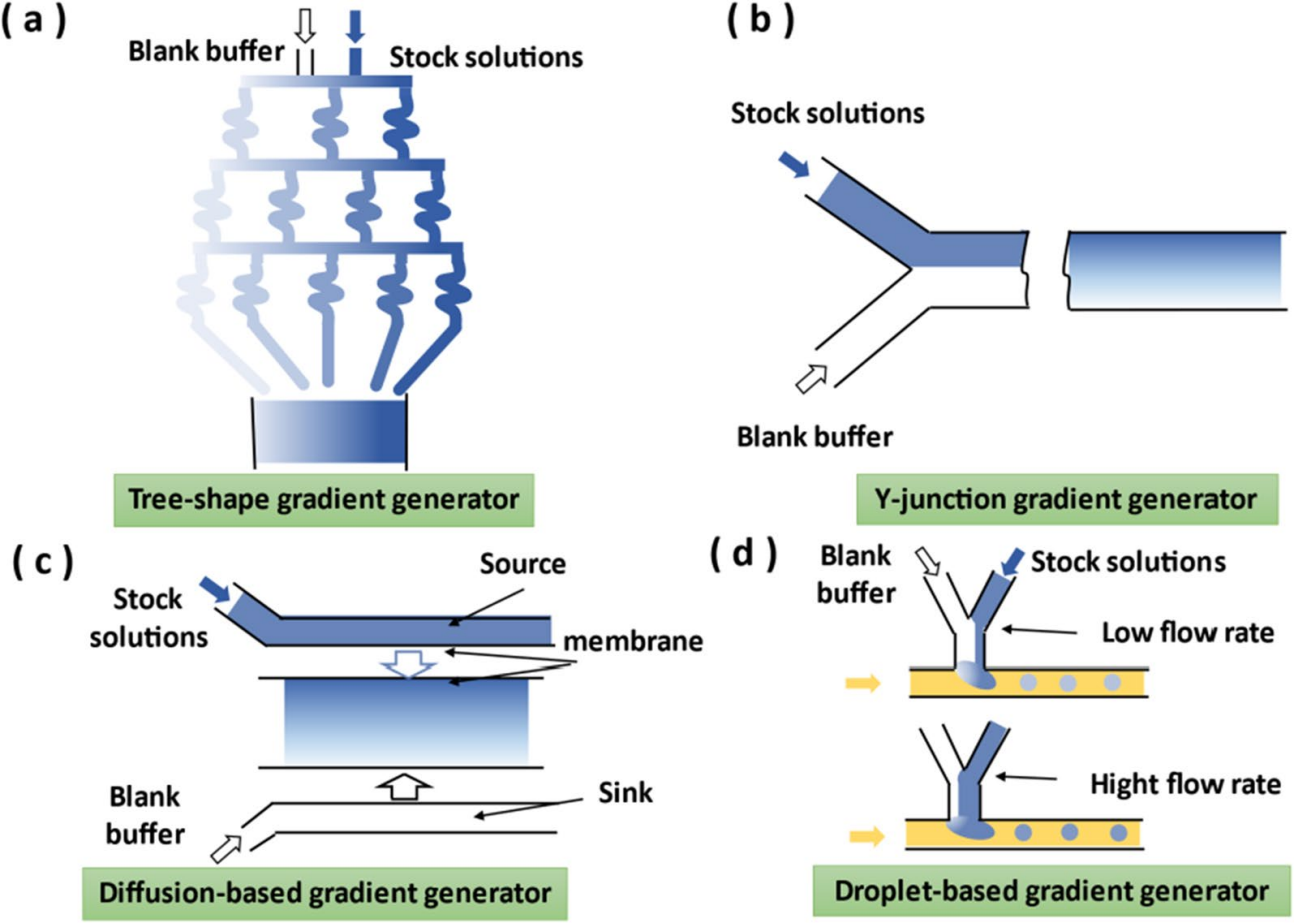
Schematic diagram of different gradient generators. **a** Tree-shape gradient generator. **b** Y-junction gradient generator. **c** Diffusion-based gradient generator. **d** Droplet-based gradient generator

Convection-based gradient generators include tree-shape networks and Y-junction. Tree-shape network is one of the earliest microfluidic gradient generators, as shown in Fig. 7a. For tree-shape network, the upstream fluid is split and flowed into the neighboring branch microchannels driven by pressure. After the fluid is fully mixed in the branch microchannels, the splitting and mixing process is repeated in the microchannel of the next layer. The final outlet forms a concentration gradient. Theoretically, the resolution of the gradient increases with the number of branches [146]. The fluid analysis of tree-shape networks can be analogized to the analysis of electronic circuits. For example, pressure drop, volumetric flow rate and hydraulic resistance correspond to voltage, current, and resistance, respectively [147]. Circuit analysis enables rapid prediction of pressure-driven laminar flow in microchannels, which is convenient for designing complex tree-shape networks prior to fabrication. Y-junction gradient generator is another kind of convection-based gradient generator. Fluids from different inlets meet at the Y-junction and diffuse in the microchannel, forming a gradient perpendicular to the flow direction (Fig. 7b). Adjusting the flow rate of different laminar flows or replacing special mixing channels can form smoother or periodic concentration gradients [148, 149].

#### Diffusion-based gradient generator

Convection-based gradient generators can rapidly form a stable gradient, but the shearing force of the flow streams may cause damage to the cells. While the diffusion-based gradient generator does not form shear force, which is conducive to cell research. The classic elements of a diffusion-based gradient generator usually include membranes, source and sink regions, and gradient-distributed microchannels [150–152] (Fig. 7c). The source region contains high concentrations of molecules, and the sink region contains only blank buffer. Molecules diffuse through the membrane, forming a concentration gradient from the source region to the sink region. At the same time the high fluid resistance of the membrane limits the fluid flow caused by the pressure difference in the system, thus avoiding the induced shearing force.

#### Droplet-based gradient generator

Droplet-based gradient generators are suitable for high-throughput microfluidic gradient analysis (Fig. 7d). Droplet-based gradient generators utilize flow focusing or T-junction type plugs to generate droplets. The concentrations inside the plug were controlled by varying the relative flow rates of the several streams of stock solutions [153]. In addition, before the droplet generation process, the stock solutions can be formed into a gradient by the above-mentioned method, and then droplets of different concentrations can be formed [154, 155].

In summary, convection-based gradient generators are suitable for generating gradients that require dynamic regulation, because the continuously flowing fluid can make the gradient generator have a faster response time. However, the shearing force is probably to damage biological samples. Diffusion-based gradient generators are suitable for generating stable gradients, which is friendly to biological samples. However, this method is slow in adjusting the gradient. Droplet-based gradient generators can provide a closed microenvironment to achieve a small shearing effect inside the droplets, and the discrete volume of the droplet can also realize the quantitative analysis. However, for droplet-based gradient generators, accurate droplet generation and control technology is necessary.

Furthermore, gradient generators mentioned above can be modularized because they can be used as independent functional modules. Insides modularized gradient generators, fluids of different concentrations and types from the upstream region have concentration gradients in the downstream region, without breaking the module connection.

### Trap

The microfluidic trap of single and cell clusters has shown great potential in promoting medical and biological research [156, 157]. In traditional cell research, cells are studied in a larger population, in which the measurement can only reflect the average value of multiple cell responses, and may ignore the valuable research of individual cell [158]. The microfluidic trap can capture the single cell and cell clusters in the controlled and stable site, which provides a method for analysis at the single cell level with the advantages of high throughput and precise control.

In generally, microfluidic traps can be categorized into contact and non-contact methods [159, 160]. The contact traps include groove or convex-based trap, inertia-based trap, microvalve-based trap and capillary-based trap, and the non-contact traps mainly consist of DEP trap, magnetic trap, acoustic trap and optical trap. Contact traps utilize mechanical barriers or obstacles to separate the cells from the fluid, and the cell will remain at the hydrodynamic capture site. Non-contact traps restrict cell movement through the force formed by external physical field.

#### Contact trap

The contact traps only rely on hydrodynamic force and do not require external energy. Groove and convex traps are typical microfluidic trapping devices [161–164]. Their pattern arrays of groove or convex (e.g., rectangles and triangles) are fabricated into microchannel. When the fluid with target cells flows through these arrays, the cells will be stuck or fall into these patterns. The flow velocity of inertial traps is usually higher than that of the groove and convex traps. The inertial trapping site is located in the expansion region next to the main channel [165–167]. When the cells flowing the main channel encounter the expansion area, because of the long distance form the main channel to the expansion region walls, the wall-induced inertial lift forces in the cells are weaker than the shear-induced inertial lift forces in the cells. Therefore, the cells are pushed from the main channel to the expansion region.

However, groove or convex-based trap and inertia-based trap are difficult to adjust the size pf target cell in real time, while microvalve-based trap can quickly change the captured cell by adjusting the diameter of the microvalve through pressure [168–170]. When cells flow through the bifurcation with two directions, the fluidic resistance in the straight channel is smaller than that in the bypass channel. A cell flows through the straight channel preferentially and then is captured on the straight channel. Once the straight channel is filled (similar to the microvalve closing to prevent fluid flow), other cells will flow through the bypass channel, enter the next bifurcation and repeat the trapping process. To study biological samples with lower concentration and biological information with more sensitive level, capillary-based traps are developed to capture nanoliter fluid [171–173]. The structure of capillary-based traps composes of a main channel and many pairs of counter traps. First, the sample is injected into the chip followed, then the main channel is purged with air to remove the residual sample, and oil is added to wrap the droplets. Each of the nanoliter droplets is captured in the trap sites.

#### Non-contact trap

For non-contact traps, the external fields typically use the electrical field, magnetic field, acoustic field, and optical field for cell trapping. DEP technology can be used to push neutral cells to the direction of higher or lower electric field density, which is depending on dielectric properties of the cells and medium. Because the highest amplitude of the electric field gradient is near the electrode, cells will be captured by the electrode array when they travel through the DEP trap [174, 175]. However, the contact between the electrode and the solution is easy to cause the solution to be electrolyzed and effect the purity of the sample. Magnetic trap does not affect the purity of the sample and does not need to be fabricated microstructure array. When magnetic particles or magnetically labeled samples flow through the magnetic trap, these particles and samples will be captured by the magnetic field gradient generated by permanent magnets or electromagnets [176–178].

In nature, most of the cells are non-magnetic. Magnetic immunolabelling of cells adds the complexity of cell trapping. The optical trap is a non-label method for cell trapping. In optical traps, optical tweezers, which utilize optical gradient force and scattering force from a highly focused laser beam, can be used to capture cells and guide them to other locations [179, 180]. The optical gradient force can pull cells toward the highest intensity region, while the scattering force can push cells along the direction of light propagation. However, continuous laser irradiation is easy to damage cells and increase solution temperature. The use of acoustic field is also an accurate and label-free trapping method, and has little damage to cells. In the acoustic trap, when the acoustic wave encounter cells, because the acoustic wave is the traveling wave, the cells are pushed along the propagation direction by an acoustic radiation force, until they are retained in the trap sites [181]. In addition, the acoustic trap can use a standing ultrasonic wave. Therefore, the cells will be pushed to the trapping nodes by a radiation force [182].

In summary, contact traps utilize hydrodynamic force and do not require external energy with the advantage of high throughput. However, contact traps often need to combine with microfabrication technology and can only capture cells in fixed sites. Compared with the contact traps, non-contact traps can flexibly choose the captured site of cells and quickly release the captured cells, due to the fast response of external physical fields. However, the need for energy sources will limit the portability of non-contact traps.

In addition, both contact traps and non-contact traps mentioned above can be modularized. Because they can be used as independent functional modules to trap cells in the fluid by their geometric structures or external physical fields, without affecting the operation of other modules and the connection of modules.

### Cell culture

In life science, cell culture in vivo involves animal experiments, which are difficult to observe and increases the complexity. Therefore, the combination of microfluidic technology and cell culture in vitro has gradually attraction scientists’ attention. In recent years, microfluidic cell culture has been applied to drug discovery, immunology, cancer research, stem cell proliferation and differentiation, tissue engineering and other applications [183–186].

In the microfluidic chip, cells can be continuously and dynamically supplied with nutrients. In this section, we mainly introduce co-culture and microenvironment modulation of microfluidic cell culture.

In a microfluidic chip, the design of co-culture can be used to research the interaction between different kinds of cells [187]. The co-culture system includes the culture of direct contact cells and the culture of spatially separated cells [188, 189]. In direct contact co-culture, two or more kind of cells are seeding on the same interface and allowed to contact directly to regulate structures, functions and behaviors of cells in the culture chamber. In indirect co-culture, different kinds of cells are usually separated by membranes, and cells communicate and influence each other through transmembrane biological factors [190, 191]. Compared with integrated microfluidics, co-culture on modular microfluidics has better flexibility and applicability. For example, when studying the co-culture of different tissues under the same conditions, it is difficult to achieve tissue maturation under different conditions and co-culture under the same conditions in the integrated microfluidic chip [6]. The modular method can make primary cells mature on different modules to form different tissues, and then these modules are connected to co-culture different tissues under the same condition.

In cell culture, in addition to the interaction of co-cultured cells, the microenvironment around cells is also an important factor to affecting the growth, development and repair of cells. The cell microenvironment consists of biochemical factors (e.g., soluble factor, extracellular matrix and gradient) and physical factors (e.g., force and temperature that can be sensed by cells) [192]. Compared with traditional integrated cell culture, microfluidic technology can be used to flexibly adjust biochemical and physical factors in the cell microenvironment in time and space [193].

In summary, co-culture mainly researches the interaction between different cells, while microenvironment modulation mainly researches the influence of the microenvironment on cell life activities. For on-chip cell culture, modules of co-culture and microenvironment modulation have more application scenarios than integrated microfluidics. Because they can independently adjust the medium concentrations and types of different culture modules, which is important for studying cells with different culture conditions.

## Connection between microfluidic modules

**Fig. 8:**
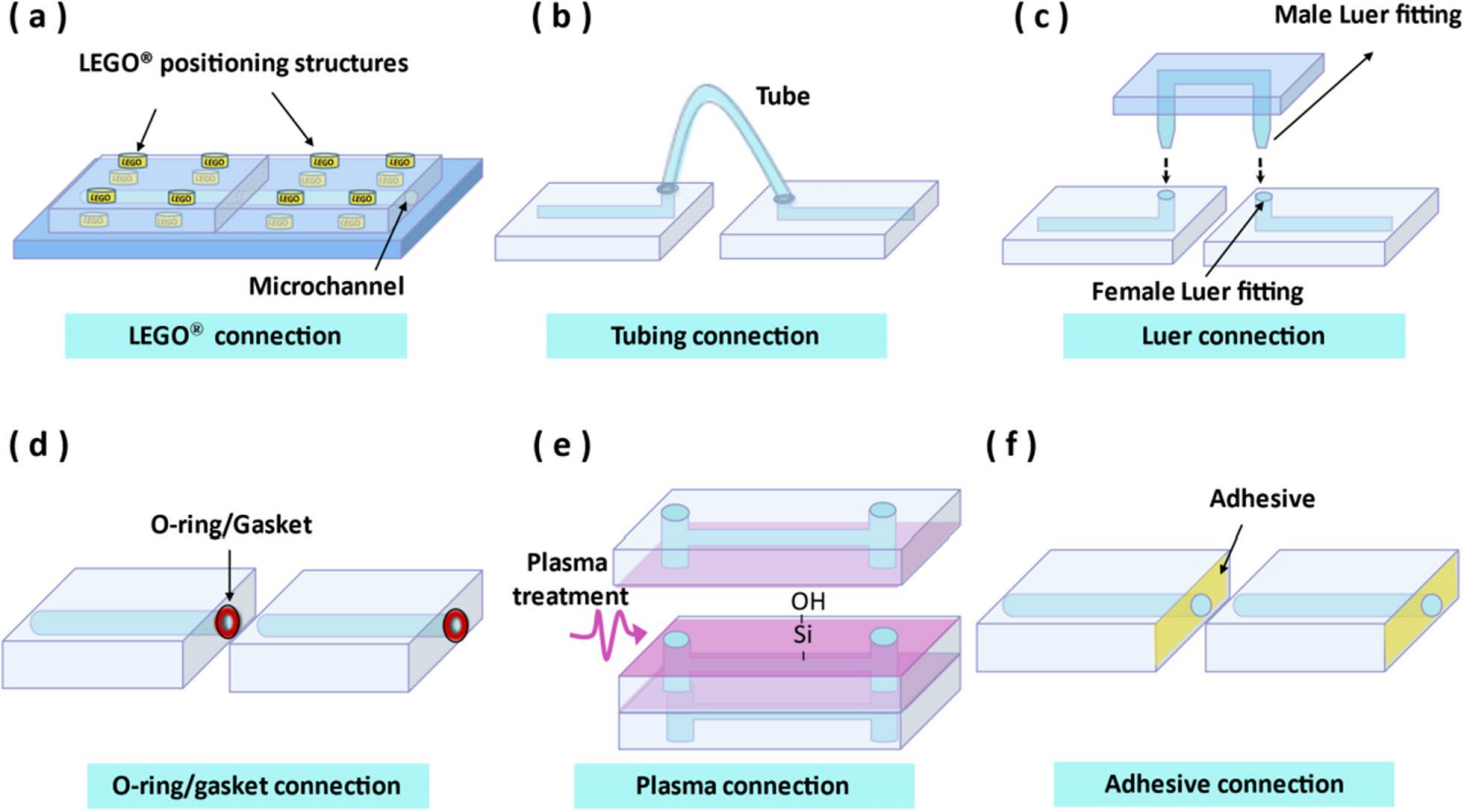
Schematic diagram of different connection methods. **a** LEGO® connection. **b** Tubing connection. **c** Luer connection. **d** O-ring/gasket connection. **e** Plasma connection. **f** Adhesive connection

**Fig. 9:**
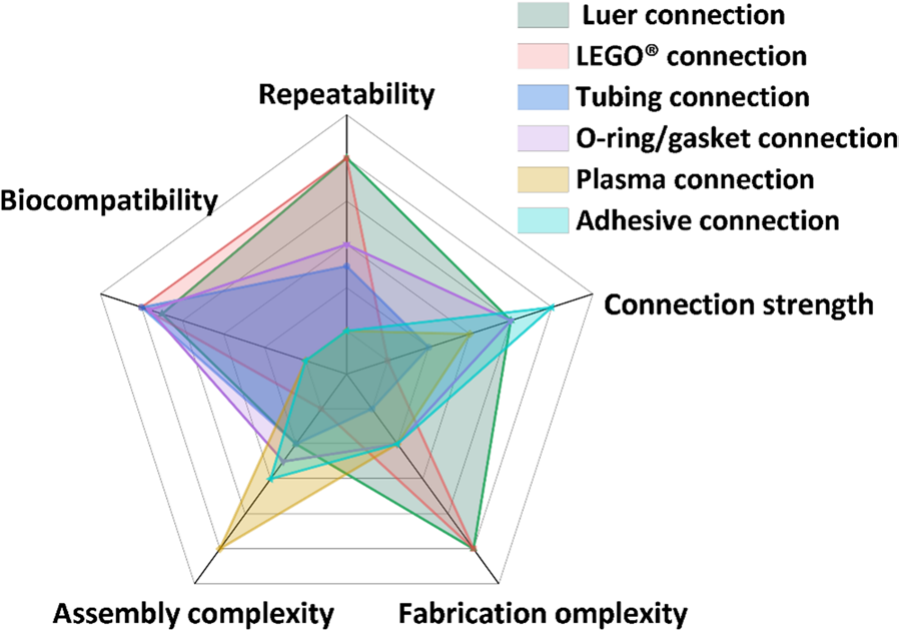
Comparison of repeatability, connection strength, fabrication complexity, assembly complexity and biocompatibility of different connection methods

To form a system, each individual microfluidic module needs to be connected. The connection of modules needs to prevent liquid leakage and ensure smooth operation of the whole system [7, 8]. So far, the connections of microfluidic modules include the LEGO^®^ connection [3], the tubing connection [161], the Luer connection [2], the O-ring/gasket connection [194, 195], the plasma connection [196] and the adhesive connection [197] (Fig. 8). Compared with the reviews from Fan et al. [7] and Lai et al. [8], this paper evaluates the properties of different connection methods, including repeatability, connection strength, fabrication complexity, assembly complexity and biocompatibility (Fig. 9).

### LEGO^®^ connection

LEGO^®^ connection derives from idea of LEGO^®^ blocks. Specifically, the researchers replicated the easily aligned and assembled LEGO^®^ interface onto the microfluidic modules. The LEGO^®^ microfluidic modules include knobs and grooves, which combine to realize the positioning, channel alignment and connection between the microfluidic modules (Fig. 8a) [3, 6, 198–204]. The LEGO^®^ connection is usually an interference fit between blocks, so it will not affect the activity of cells or bacteria in the module. Ideally, modular microfluidics can be freely assembled into a system according to desired functions, and can be disassembled and reconfigured into other systems. For example, Vittayarukskul et al. [3] proposed a LEGO^®^-like modular microfluidics platform, and their microfluidic modules can be fastened and stacked on a traditional LEGO^®^ board to realize 3D microfluidic networks. The spacing of knobs and grooves is strictly equal, and the channels of the modules are also processed in the same position. Thus, the positioning of knobs and grooves allows automatic alignment and connection of the channel, when the module is assembled. As a result, an interference fit was formed at the connections during block assembly, which can resist block displacement due to vertical and lateral compression and maintain the pressure sealed of the bottom and lateral interfaces. However, the burst pressures of this platform are small due to interface shifting under pressure, block edge lift and high surface roughness, from 0.11 psi to 0.13 psi. To improve sealing performance at block junctions, Langelier et al. [199] modified the connection surface of the block with jigsaw-like lock and key (male and female) structures, which enables the microfluidic system to withstand a burst pressure of up to 15 psi.

In summary, the LEGO^®^ connection has a compact system volume, less dead volume, and excellent repeatability and biocompatibility. In addition, the LEGO^®^ connection has simple assembly due to its alignment structure and assembly reversibility. However, the LEGO^®^ connection has complex fabrication due to the design requirements of knobs and grooves, and has weak connection strength. Therefore, the LEGO^®^ connection is more suitable for the liquid with lower pressure.

### Tubing connection

The tubing connection is the most straightforward and widely-used method in modular microfluidic systems [144, 161, 205–224]. The inlet and outlet ports of each microfluidic module are predefined by punching or drilling through holes to the microfluidic channels so that fluids can be infused in or extracted out through the ports. The interference fit between the tube and the inlet/outlet of the module will not affect the activity of cells or bacteria in the module when studying life science. Then, flexible plastic tubes connect the corresponding ports of adjacent microfluidic modules so that the fluids from the upstream module can enter the downstream module continuously (Fig. 8b). For example, Sun et al. [144] proposed a modular microfluidic scheme to achieve real-time gradient generation. They have designed several basic modules such as collectors, combiners and distributors. Thus, the end-users, such as biologists and chemists, only need to connect these modules with tubes according to the predesigned scheme instead of going through the whole fabrication process.

In summary, the tubing connections have simple fabrication, simple assembly, excellent biocompatibility and good repeatability. However, with the increase of the times of the plugging, it is easy to cause the chip inlet and outlet to crack, leading to the decline of connection strength and repeatability. Furthermore, the tubing connection consumes more working liquid due to a large dead volume in the connecting tubes, and is prone to cause more particles agglomeration compared with LEGO^®^ connection.

### Luer connection

In the Luer connection, a Luer taper is a standardized microvolume free-leakage connector that is connected by a male Luer fitting and a matching female Luer fitting part, which is widely used in medical and laboratory instruments [2, 194, 195, 204, 225–230] (Fig. 8c). For example, Yuen designed a “plug-n-play” modular microfluidic system for biological and chemical applications [2]. The female Luer fitting is interconnected with the channel in the connector, and the male Luer fitting is interconnected with the channel on the functional module. When the connector is placed on the positioning groove of the motherboard according to the pre-designed scheme, the modules with different functions are placed and assembled to the connector. The pairing of the male and the female Luer fitting causes the fluid to pass through the channels of the Luer fitting from the upstream module to the downstream module, which can withstand the burst pressure as high as 51 psi. Similar to LEGO^®^ connection, the connection of male and female Luer fitting will not affect the survival of cells or bacteria. The difference from LEGO^®^ connection is that the Luer taper is usually used as part of the channel to locate and interlock to microfluidic modules.

In summary, the Luer connection has excellent biocompatibility, simple assembly, good connection strength for the liquid with higher pressure and excellent repeatability for multiple assembly and disassembly. However, Luer taper requires delicate design and processing because design and processing of the connectors that mate with each other is an important indicator of no leakage.

### O-ring/gasket connection

The rubber O-ring/gasket placed at the joint, due to its flexibility, can deform appropriately to connect the channels on adjacent modules with slight differences [200, 231, 232] (Fig. 8d). The production of microfluidic modules is difficult to ensure that the sizes of channels are identical in high precision. Besides, it is difficult to avoid the problem of slight misalignment during the assembly process of modules. Therefore, the elastically deformed O-ring/gasket can be added at the joint to prevent leakage caused by small errors in channel size or matching alignment. For example, Lee et al. [194] proposed 3D-printed modules for integrated microfluidic devices. They positioned individual microfluidic modules with metal pins, and connected channel junctions with greased O-rings, pristine O-rings, and no O-rings at all, respectively. The results showed that greased O-rings were the best at connecting, withstanding fluid pressures of more than 29 psi because the hydrophobicity of the grease acts as a natural barrier to minimize solution penetration around the interconnected area. Gaskets are similar in construction to O-rings, but generally have a larger sealing area [205, 233]. Zhou et al. [195] designed a plug-and-play modular microfluidic emulsion production system that uses a combination of threads and gaskets to seal joints. The gasket is strained and squeezed by the threads, then fully attached to the glass capillary and fills the thread gap to avoid fluid leakage. Because O-ring and gasket are usually non-toxic elastic materials, cells or bacteria will not be injured when contacting with them.

In summary, O-ring and gasket generally have excellent biocompatibility and good repeatability for life science, simple fabrication and assembly, and good connection strength for the channel operating under the higher pressure.

### Plasma connection

In the vacuum state, the high-frequency generator ionizes the gas to generate plasma for surface activation and modification of microfluidic modules, which enhances the adhesion of modular interfaces and realizes bonding [196, 228, 234–240] (Fig. 8e). Warkiani et al. [241] proposed an inertial microfluidic system about membrane-less microfiltration. Each layer of PDMS modules contained four systematically connected spiral channels. The PDMS modules were treated with an oxygen plasma machine to connect and seal the inlets and outlets. Compared with the traditional integrated microfluidic system, this multiplexed microfluidic system can vertically stack multi-layer PDMS modules to reduce the occupied area, and can be customized by end users to achieve high-throughput separation according to their needs. However, when cells are cultured in the modules, these culture modules cannot be connected by plasma treatment because plasma on cells will damage cell membranes.

In summary, the plasma connection is an irreversible connecting method due to the strong chemical bond formed between the connection surfaces. Putting the module into the plasma cleaner is easy to produce chemical bond on the module surface. However, connecting with plasma treatment requires a relatively short operation time (about several minutes) before the treated surfaces lose their reactivity, which may be inconvenient for the assembly of the microfluidic module for the end users. In addition, the plasma connection has poor biocompatibility.

### Adhesive connection

The adhesive connection is manly to add adhesive agents or adhesive films at the connection surface of modules to make the modules interlock by the adhesive force (Fig. 8f). In generally, adhesive agents can be used to connect rigid microfluidic modules [199, 242–249], and adhesive films can be used to connect soft microfluidic systems such as skin-interface [250]. For example, Rhee et al. [197] designed microfluidic assembly blocks and compared different connecting methods of microfluidic blocks, including direct mechanical connecting and separately adding adhesive agents, such as a mixture of PDMS prepolymer and curing agent (10:1), PDMS curing agent and UV-curable glue. The results show that the reversible mechanical connecting can maintain inside pressures of 4–6 psi, while the adhesive connecting can maintain inside pressures greater than 30 psi. However, adhesives are usually toxic, which will affect the activity of cells and bacteria.

In summary, the advantages of Adhesive connecting are simple fabrication and assembly, excellent connection strength and long-lasting connection time [251, 252]. However, although using adhesive agents or films to seal the connection surface between modules can improve the working pressure of liquids, adhesive agents or films can affect the disassembly and cleaning of the modules, which reduces the recyclability of modules. In addition, the adhesive connection has poor biocompatibility. Thus, adhesive connecting is a suitable choice when end users require a long-term or disposable microfluidic system without reversible disassembly and cell culture.

## Applications

Microfluidic devices have been used for biomedical research due to their advantages of small reagent consumption, large surface area and small footprint. The modular microfluidic technology further lowers the barrier for using microfluidics, and provides flexible and customizable systems for end users without expertise in microfluidics. Here, we elaborate on some typical applications of modular microfluidics in life science, including cancer cell isolation, polymerase Chain Reaction (PCR), Organs-on-a-chip, biomarker research and particle synthesis, as summarized in Table 1.

**Table 1:** Typical applications of modular microfluidics in life science

Application	Modules	Connection	Specific applications	Refs.
Cancer cell isolation	Spiral separators	Plasma connection	Separation of breast cancer cells and lung cancer cells, respectively	[196, 234]
	Spiral separators and serpentine separators	Adhesive connection	Separation of breast cancer cells and lung cancer cells, respectively	[251]
	Flow regulators and spiral separators	Plasma connection	Separation of breast cancer cells and lung cancer cells, respectively	[240]
	Spiral separators and serpentine separators	Plasma connection	Separation of colon cancer cells, lung adenocarcinoma cells and renal clear cell carcinoma cells, respectively	[236]
	Serpentine separators	Tubing connection	Separation of breast cancer cells	[209]
PCR	DNA extraction modules, storage of lyophilized reagents modules, a PCR module and a hybridization module	Tubing connection	Identification of bacteria	[217]
	PCR reactors, micromixers and LDR reactors	Adhesive connection	Point mutation identification of human genome	[244]
	PCR chambers and a Peltier device	Adhesive connection	Detection of SARS-CoV-2	[245]
Organs-on-a-chip	Heart, fat and liver organ chips	LEGO^®^ connection	Formation of cardiac tissue	[204]
	GI tract organ chips and liver organ chips	LEGO^®^ connection	Simulation of metabolic activity and response to toxic substances	[6]
	Heart, liver and lung organ chips	Tubing connection	Drug administration	[218]
	Heart and liver organ chips	Tubing connection	Monitoring of drug-induced organ toxicity	[213]
	3D culture chips, 2D culture chips, pumps and reservoirs	Magnetic adsorption	Modeling systemic bioactivation of nutraceuticals and prodrugs	[253]
Biomarker research	Pumps, valves, mixers, sensors and heaters	Adhesive connection	Detection of metal ions and cancer protein biomarkers	[246]
	Mixing modules, an IEX module and a SEC module	Tubing connection	Purification of protein	[211]
	Valves, PMMA chambers and porous membranes	UV treatment and Tubing connection	Sorting of small peptides from bigger proteins	[215]
	PMMA chambers	Tubing connection	Quantitative assessment of metabolites	[219]
	Sample injection modules, cell culture modules and detection modules	Tubing connection	Quantitative assessment of metabolites	[220]
	Heat exchanger	Adhesive connection	Assay of calcium signaling	[254]
Particle synthesis	Tesla microvalves	Plasma connection	Synthesis of chitosan nanoparticles	[239]
	Pumps, valves, vortex-type mixers, reservoirs, and heating blocks	Luer connection	PCR and synthesis of gold nanoparticles	[227]
	Co-flow, counter-current flow focusing and three-phase flow modules	Tubing connection	Synthesis of gold nanoparticles, liposomes, piroxicam monohydrate crystals and silver nanoparticles	[222]
	Tube-in-tube contactors, regulators, pumps and coiled millireactors	Tubing connection	Synthesis of iron oxide nanoparticles	[214]
	T-shaped droplet generators, single, two parallel and two sequential inlet modules	Gasket and luer connection	Synthesis of magnetically responsive microparticles	[195]
	Parallel droplet generators	Adhesive connection	Synthesis of chitosan/TiO2 composite microspheres	[247, 248]

### Cancer cell isolation

Cancer is the leading cause of death in the world, 90% of which is caused by metastasis of solid tumors [255]. Therefore, early diagnosis of cancer disease in asymptomatic populations can reduce tumor deterioration and metastasis by providing effective intervention and treatment [256]. Isolating circulating tumor cells (CTCs) from the peripheral blood of patients emerges as a promising method as a “liquid biopsy” for cancer early diagnosis, prognosis and personalized therapy [257]. However, CTCs are extremely rare (1–100 CTCs per mL of blood) at the early stage of cancer disease, consisting of only a few out of one billion cells in the blood, making their isolation and characterization an extreme technological challenge. Multiple separations can improve separation efficiency, but it is difficult for an integrated microfluidic system to adjust the number and combination of separators according to the requirements of separation efficiency and purity. The customizable modular microfluidics is a promising solution, since it can combine and integrate multiple modules to amplify processing throughput and enhance separation purity.

Microfluidic modules in parallel can improve the throughput and maintain a small footprint. Warkiani et al. [196, 234] stacked up three spiral microfluidic chips to isolate CTCs, and the processing throughput of this stacked device is as high as 0.75 mL/min for the whole blood. The clinical use of this microfluidic chip was verified by detecting CTCs from blood samples of patients with advanced-stage metastatic lung and breast cancers with 100% separation accuracy. Similarly, Fang et al. [251] designed a stacked and miniaturized inertial microfluidic centrifuge with a washing module and a concentrating module. In the cellular flow rates of 0.4 mL/min, they used this microfluidic centrifuge to extract MCF-7 breast cancer cells from the culture medium, and to extract A549 lung cancer cells from the calcein-AM solution with solution exchange rates of about 95% and 98%, respectively.

Microfluidic modules in series are an alternative method for improving the throughput. Jiang et al. [240] proposed a microfluidic handheld cell sorter (μHCS) for high-throughput tumor cell sorting, and the core microchip of μHCS is stacked up by two flow regulators and a spiral separator. The flow regulators could achieve dynamical flow regulation according to the requirement for the downstream. In the total flow rate range of 1.1 to 1.2 mL/min, the spiral separator can separate the MCF-7 breast cancer cells and A549 lung cancer cells from blood cells at about 85% and 83% recovery ratios, respectively. Furthermore, the μHCS can also be used to separate the rare malignant tumor cells (MTCs) from patients’ pleural effusions with metastatic stage IV non-small cell lung cancer (NSCLC). Similarly, Xu et al. [236] stacked a spiral channel module and two different square serpentine channel modules, which is used for the high flow rate input of upstream samples and the low flow rate output in convenient downstream detection. At a high inlet flow rate of 1.3 mL/min, they successfully separated three tumor cells (SW480, A549, and Caki-1) from massive background cells with the separation efficiency of > 80% and the separation purity of > 90%.

Cascading multiple modules is an alternative way to further improve the separation efficiency of cancer cells. Cha et al. [209] developed a cascaded microfluidic separation module by combining two sinusoidal channel modules to purify breast cancer cells from the blood samples consecutively. The working flow rate range of the second microfluidic module was regulated by embedding periodic obstacle microstructures in the sinusoidal channels, so that the output flow rate of the upstream microfluidic module could match the required input flow rates for the downstream microfluidic module. The results show an excellent separation performance. For the samples with the ratio of primary cancer cells of 0.01% and 0.001%, the purity was increased by 3 to 4 orders of magnitude.

### PCR

PCR is a molecular biological technology to amplify specific DNA fragments by controlling the temperature cycle, which has been widely used in diagnosis analysis of pathogenic bacteria, viruses and gene mutation detection, as well as clinical diagnosis [258, 259]. However, for complex assays, it is difficult for integrated PCR microfluidics to accurately control and maintain the temperature of different areas on a single plate [260]. In addition, the integrated PCR microfluidic systems still have challenges for different field deployments. Modular PCR microfluidics allows the development and optimization of individual assay steps, which is a promising direction because it can freely expand more functional modules and adjustment rapidly onsite microfluidic functions.

In clinical diagnosis analysis, modular PCR microfluidic systems can be assembled by individual PCR analysis modules for detecting bacterial and human gene mutation. For diagnostic assays of bacteria, Hlawatsch et al. [217] developed a modular microfluidic platform including DNA extraction module, storage of lyophilized reagents module, a PCR module and a hybridization module. As proof of concept, the performance of this platform was assessed by the assay and the identification of *Neisseria meningitidis* bacteria, as low as 1000 cells/mL. In addition, the combination of PCR and ligase detection reaction (LDR) is a common technique for the detection of human gene mutation [261, 262]. Lee et al. [244] designed a modular microfluidic platform that can vertically stack modules with different functions. As proof of concept, they respectively assembled a three-module (PCR reactor, micromixer and LDR reactor) and two-module (micromixer and LDR reactor) microfluidic devices for point mutation identification of human genome.

In addition to clinical diagnosis analysis, onsite detection and rapid diagnosis of pathogens are a promising trend of PCR. Severe acute respiratory syndrome coronavirus 2 (SARS-CoV-2) has been spreading worldwide, seriously affecting people's health and even causing death. Reverse transcription (RT)-PCR is the gold standard for the identification of SARS-CoV-2 because of its high selectivity and sensitivity [263]. Nguyen et al. [245] developed LEGO^®^-like RT-PCR and RT-loop mediated isothermal amplification (RT-LAMP) platform for the rapid onsite diagnosis of SARS-CoV-2, in which the microfluidic chip mainly consisted of serval PCR chambers and a Peltier device. They used endpoint RT-PCR and real-time PCR to demonstrate the excellent screening and confirmatory abilities for SARS-CoV-2 samples, shortening the diagnosis time to an hour and halving the amount of reagent required, respectively with 100% sensitivity and 100% specificity. In addition, this system can also be used for colorimetric RT-LAMP assay to identify positive and negative RNA extracts with 100% specificity.

### Organs-on-a-chip

Organs-on-a-chip devices are used to accurately mimic the natural physiology and function of organs, which can reduce experimental costs, reduce animal experiments and improve the success rate of experiments [264, 265]. Besides, organs-on-a-chip devices enable researchers to clearly see the functions, behaviors and responses of organs at the cellular and molecular levels. However, the integrated multi-organ microfluidic platform is difficult to independently adjust the tissue maturation conditions and drug screening conditions of each simulated organ in a single plate. The combination of multi-organ chips and modular microfluidic technology can independently adjust the cultivation conditions and study different drug screening of each organ module according to the requirements of the end user [5, 216].

Modular organs-on-a-chip devices allow tissues to mature on a single chip and connect several chips to study the performance of a multi-organ system. Loskill et al. [204] designed a plug-and-play microfluidic system including heart, fat and liver organ modules. To verify the ability of this system, they separately cultured induced pluripotent stem cells (hiPSCs) into two heart modules and then connected these heart modules to form micro-physiological tissues. The mimic tissues present homogeneous beating with physiological beat rates. Similarly, Mandy et al. [6] presented a co-culture organs-on-a-chip platform by stacking a gastrointestinal (GI) tract chip and a liver chip. The measurement results of urea, albumin and cytochrome P450 (CYP) enzyme show that the liver tissue maintained high levels of metabolic activity and the capacity to respond to toxic substances.

Modular organs-on-a-chip can be used to study multiple drug screening. Skardal et al. [218] obtained a three-tissue organ-on-a-chip platform consisting of heart, liver and lung organ chip. They demonstrated the value of inter-tissue interaction to drug (e.g., propranolol, epinephrine and bleomycin) administration. Similarly, Zhang et al. [213] used an organ-on-a-chip platform for monitoring drug-induced organ toxicity. Specifically, they respectively used a liver-and-heart-on-a-chip insulted with acetaminophen (APAP) for up to 5 days, and a liver-cancer-and-heart-on-a-chip challenged with doxorubicin for up to 24 h, to research the responses of chronic drug and acute toxicity. In addition, Ong et al. [253] presented a Tetris-Like (TILE) modular microfluidic system for truly mimicking multi-organ interactions, and then successfully emulated liver-mediated bioactivation of a nutraceutical compound and a cancer prodrug.

### Biomarker research

Biomarkers can reflect the changes in cells or molecules related to diseases in the human body. The reliable screening and detection of biomarkers are crucial for clinical diagnosis and treatment. The usual research methods of biomarkers include proteomics, metabolomics and so on [266]. Microfluidic technology can accurately manipulate fluids of microliter and even nanoliter volume with high throughput, which matches the volume level of cells or molecules (micrometer and nanometer), so it becomes an effective tool for biomarker detection. However, when an integrated microfluidic system is used to study multiple biomarkers, it is difficult to flexibly adjust the functions of the chip for different biomarkers. The modular method can utilize standardized functional modules and flexibly replace/rearrange modules according to experimental purposes.

Proteins and peptides are considered as critical biomarkers of diseases. Shaikh et al. [246] demonstrated a multiple-chip module (MCM) system with double layers. The bottom layer is a microfluidic breadboard (FBB) with many individual modules including mixer, valve, pump, sensor and heater. In contrast, the top layer is microfluidic connecters, which can freely connect the individual modules in the bottom layer. They verified the system capability for various biochemical applications by applying different top layers on an FBB to detect protein biomarkers of cancer and metal ions, respectively. In addition, Millet et al. [211] presented a modular microfluidic platform for protein purifications, consisting of a mixing module, an ion exchange (IEX) module and a size-exclusion chromatography (SEC) module in series. Finally, the platform yields 4.6 mg/mL enhanced green fluorescent protein (eGFP) concentrated out of 112 μL *Escherichia coli* (*E. coli*) lysate. In addition to protein detection, the modular microfluidic chip can detect the peptides. Perozziello et al. [215] used tubes to connect syringe pumps, valves, and a microfluidic chip stacked by two layers of poly-methyl-methacrylate (PMMA) and a porous membrane to form a modular peptide filtration system. The peptides expressed by breast cancer cells were filtered and deposited on a superhydrophobic surface, followed by Raman spectroscopy detection.

Substrates and products of metabolic processes can also quantify real biological reaction processes. Conventional sequential one-pot incubations cannot generate measurable amounts of hepatic phase II metabolites, so Kampe et al. [219] designed a modular compartmentalized microfluidic chip that can be used for fluidic coupling of phase I and II enzymatic transformations and the detection of metabolites. Specifically, they chose the antitussive drug dextromethorphan (DEX) as a substrate, and filled two compartments with enzyme-coated microbeads bearing CYP2D6 and UGT2B7. Finally, they quantified several metabolites with LC-MS [2] analysis to research the metabolism processes. Similarly, Munshi et al. [220] used a modular microfluidic system composed of a sample injection module, a cell culture module and a detection module in series. They electrochemically quantitated the release of nitric oxide (NO) from endothelial cells after adenosine triphosphate (ATP) stimulation. In addition, Zhu et al. [254] designed a modular and reconfigurable convective heat exchanger for temperature control of microfluidic systems. The heat exchanger comprises polymer tubes wrapped around a plastic pole and fully embedded in an elastomer block, which can be easily installed on different microfluidic structures. Then they monitored their intracellular calcium level ([Ca^2+^]_i_) under temperature cycling by using the modular heat exchanger.

### Particle synthesis

Micro/nanoparticles have shown great application potential in drug delivery and biochemical assays [224, 267–269]. The physical and chemical properties of particles mainly depend on their size and shape. Therefore, it is significant for researchers to synthesize particles with controllable sizes and shapes. The traditional particle synthesis is carried out in large reactors, which has the disadvantages of varying particle size and poor batch repeatability. Particle synthesis on microfluidic devices has the advantages of narrow size distribution, uniform shape, enhanced reproducibility, and high encapsulation efficiency [270]. However, the integrated microfluidic system is usually suitable for single-particle synthesis. Readjusting parameters and remanufacturing entire chips will consume lots of time and money, while modular particle synthesis systems provide more convenient combinations and wider usefulness. The modular microfluidic system applied to particle synthesis is categorized into the single-phase continuous fluid system and the multiphase droplet system.

In a single-phase continuous fluid system, multiple mutually soluble fluids are precisely controlled in sequence to meet and mix to form single-phase fluid. The synthesis of particles occurs in the continuous flow of single-phase fluid, which ensures the uniformity of the entire reaction conditions. Guo et al. [239] used a stacked 3D micromixer which is integrated with four Tesla microvalves to synthesize Chitosan (CS) nanoparticles. Compared with the conventional magnetic stirring method, this modular micromixer can accurately synthesize smaller and more uniform nanoparticles with higher efficiency. Moreover, Hsieh et al. [227] designed a Lego^®^-like modular microfluidic platform include pumps, valves, vortex-type mixers, reservoirs, and heating blocks. They used these functional components to assemble two devices for gold nanoparticles synthesis and PCR detection, respectively.

In a multiphase droplet system, two or more immiscible fluids meet and shear to form small droplets as separate reaction chambers, which reduces the risk of mutual contamination and improves the repeatability of synthesis. Bandulasena et al. [222] presented reconfigurable, versatile and self-alignment capillary microfluidic devices that consisted of coaxial assemblies of glass capillaries held between two plastic blocks. Then they assembled three different devices to generate micro/nano-materials, including a two-phase co-flow device for the preparation of liposomes and gold nanoparticles, a two-phase counter current-flow focusing device for the preparation of water/oil emulsions, gel microparticles and drug microcrystals, and a three-phase flow device for the preparation of multiple emulsions and silver nanoparticles. In addition to non-magnetic particles, magnetic particles can be used in magnetic hyperthermia for cancer treatment [271]. Panariello et al. [214] used a tube-in-tube gas–liquid contactor and a coiled milli-reactor to assemble four different reactor systems (single-phase and two-phase flow) for the synthesis of iron oxide nanoparticles with different characteristics. Similarly, Zhou et al. [195] presented a plug-and-play microfluidic device for versatile emulsion generation, composed of a top module for the dispersed phase supply, a glass capillary for emulsion generation and a bottom module for the continuous phase supply. They used this device to produce magnetically responsive microparticles with narrow size distribution. Compared with conventional microfluidic devices, this system provides more flexibility, since each part can be easily replaced. In addition, Huang et al. [247, 248] stacked ten droplet generator modules to form a multi-channel parallel microfluidic device for generating controllable particles with high throughput. When the throughput was 480 mL/h, they generated Chitosan/TiO2 composite microspheres with an average particle size of 539.65 µm and a CV value of about 3.59%.

## Discussion and perspectives

This paper studied the basic principles and the feasibility of microfluidic modules, as well as the connection methods and applications of modular microfluidics for life science. We first introduced the basic mechanisms of several basic microfluidic modules, including microvalve, micropump, micromixer, separator, droplet generator, gradient generator, trap and cell culture. We evaluated their feasibility as modular microfluidic components. Then, we summarized the advantages and disadvantages of six common connection methods (LEGO^®^ connection, tubing connection, Luer connection, O-ring/gasket connection, plasma connection and adhesive connection) to facilitate readers in selecting appropriate connection methods according to their requirements. Furthermore, we summarized the advantages of modular microfluidics over integrated microfluidics in biological applications including cancer cell isolation, PCR, organs-on-a-chip, biomarker research and particle synthesis. Compared with integrated microfluidics, modular microfluidics can freely select the number and arrangement scheme of modules, optimize individual analysis steps, accurately adjust the local region conditions of the system, and can be used for multiple experimental objects. Although modular microfluidics has these advantages in application, there is still a considerable gap to be filled to enable modular microfluidics to be as mature as commercial products.

The main challenge of modular microfluidic promotion is that modular microfluidics needs to solve the problems of standardized connection for module-to-module and a standard protocol for module-to-world [272]. On the one hand, the standardized connection between modules needs to match the module properties, such as the dimension of connectors and fluid pressure threshold. On the other hand, module-to-world requires to reserve the common industrial interfaces on the module, which helps modular microfluidics quickly match current industrial equipment and connection protocols. Besides, module-to-world also needs to establish the standard parameter of the entire modular microfluidics system for users and other researchers to evaluate and refer, such as working environment (e.g. temperature, humidity, etc.) and validation (number of measurements, errors, etc.). The document of ISO standards (ISO 22916:2022) has been published, specifying requirements for seamless integration with other microfluidic components and systems to facilitate the process of designing new microfluidic devices. In the future, cooperation between industry and academia is needed to provide standards for modular microfluidics and promote the industrialization and internationalization of modular microfluidics [273].

In Fig. 9, we summarize the disadvantages of different connection methods. In fact, the materials used to fabricate modules will limit the application of modular microfluidic technology. For example, the cell culture chips made of silicon elastomer is easy to absorb liquid medium and swell due to the porosity and permeability of the material. When the photosensitive resin is used to fabricate a cell culture module, it can easily affect cell activity due to its low permeability. In addition, microfluidic modules printed using thermoplastic resins should avoid specific organic solvents to prevent chips from dissolving. Therefore, when carrying out specific experiments and applications, material limitations and disadvantages of connection methods should be considered.

**Fig. 10:**
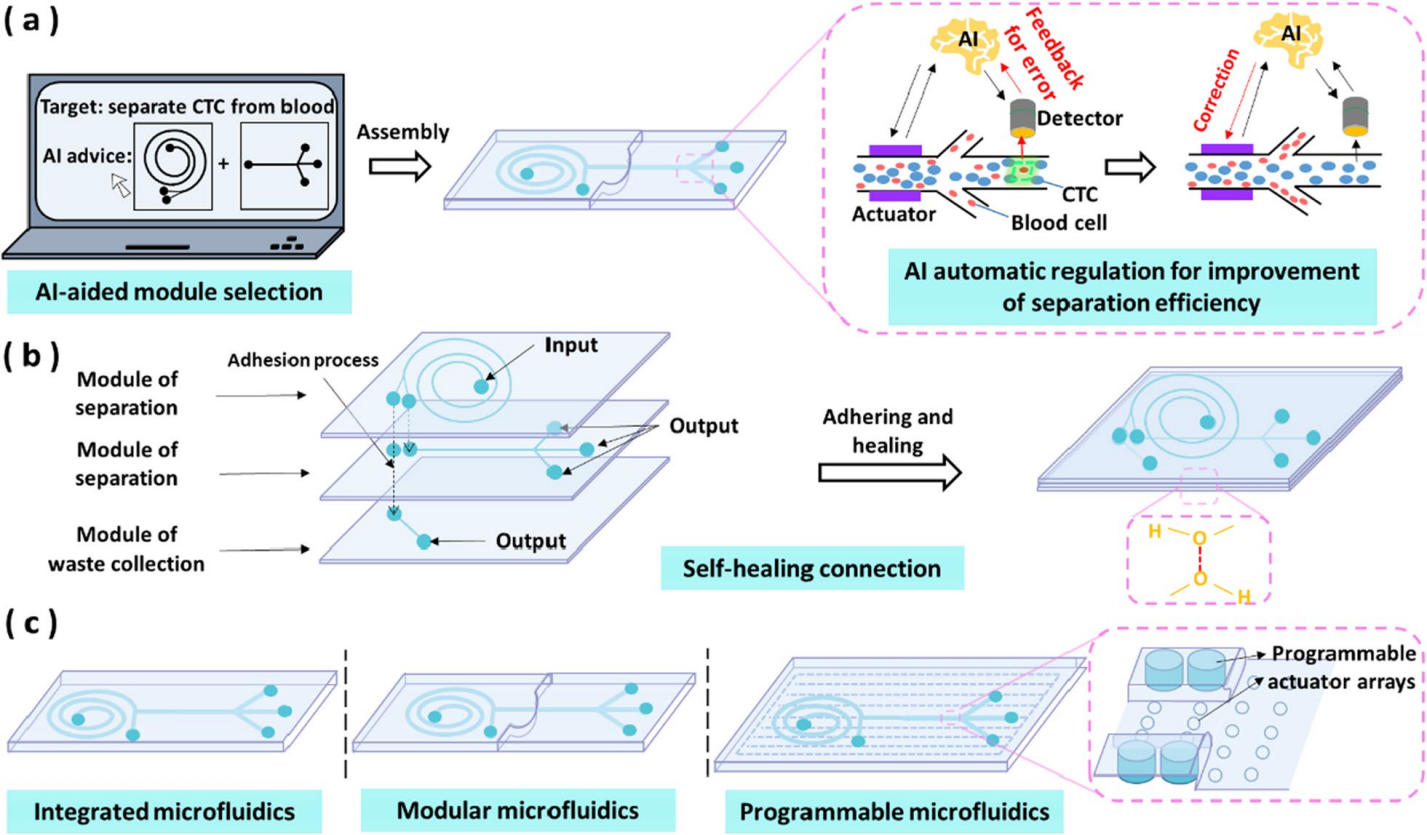
Perspectives for modular microfluidics. **a** AI-aided module selection and decision-making. **b** Self-healing connection method. **c** The concept of integrated microfluidics, modular microfluidics, and programmable microfluidics

In addition, we also look forward to several development directions of modular microfluidics. Artificial intelligence (AI) may guide the construction scheme of microfluidic modules and automated fluid control (Fig. 10a). Due to the advantages of high throughput of microfluidics and AI efficient analysis, microfluidics has begun to combine with AI technology for massive data processing [274, 275]. Besides data processing, combining modular microfluidics and AI could help end users efficiently select the modular modules, automatically control fluid in the assembled system and constantly optimize experimental parameters [276, 277]. Users can input the required functions and parameters and AI could quickly provide the assembly scheme of microfluidic modules. Furthermore, when the working fluid flows through the modular microfluidic system, the synergetic combination of IoT or LAN controllers with AI can automatically adjust the actuators (e.g., active separators) to enhance the performance of fluidic manipulation [278].

Advanced materials for microfluidic modules provide a new connection method. From the above-mentioned connections, the modular microfluidics using the tubing connection causes much dead volume of liquid, while other connection methods still require additional processing (e.g., adding adhesives or plasma treatment). Using advanced materials (e.g., hydrogels) with self-adhesive, self-healing and elastic properties as raw materials for microfluidic modules can provide a more convenient connection strategy [279–282]. Due to their self-adhesive property, modules made of advanced materials can be directly spliced with the help of the alignment structures. This method is similar to LEGO^®^ connection, but smart materials can achieve better sealing performance and even more free configuration because of their self-healing property [283] (Fig. 10b). In addition, the excellent elasticity offers the extensibility for the module to adjust the length and diameter of channels [284].

Programming the microfluidic channel will save the end users from the microfluidic fabrication process and improve the scalability and customization of the microfluidic system (Fig. 10c). For end users, although developers can develop many individual functional modules, the microfluidic modules may need the tiny modulation on size and geometry in the real-world applications. Because programmable microfluidics can program channels to freely define chip functions by developing an addressable actuator array [285], it will be a promising direction for microfluidic function configuration.

In conclusion, modular microfluidics has obvious advantages in customized functions and assembly schemes compared with integrated microfluidics. Although there are many significant advances in biological applications, modular microfluidics is still in the early stage of development and there is still a wide range of exploration space in standardization and commercialization. In addition, the modular microfluidic system can use AI to provide users with module selection schemes and automatically adjust the actuators in the microfluidic system to achieve targets; modules made of smart materials can directly contact each other to form chemical bonds on the connection surface to realize the self-healing connection, which has the advantages of solid connection strength, less dead volume, and simple manufacturing and assembly process; programmable microfluidics can form customized channels and functions through the response of the actuator on the chip. There are promising directions for modular microfluidics.

## Data Availability

All data generated or analyzed during this study are included in this manuscript and its additional file.

## References

[1] Whitesides GM (2006). The origins and the future of microfluidics. Nature.

[2] Yuen PK (2008). SmartBuild—a truly plug-n-play modular microfluidic system. Lab Chip.

[3] Vittayarukskul K, Lee AP (2017). A truly lego (R)-like modular microfluidics platform. J Micromech Microeng.

[4] Liu D (2022). Integrated microfluidic devices for in vitro diagnostics at point of care. Aggregate.

[5] Picollet-D'hahan N, Zuchowska A, Lemeunier I, Le Gac S (2021). Multiorgan-on-a-chip: a systemic approach to model and decipher inter-organ communication. Trends Biotechnol.

[6] Esch MB, Ueno H, Applegate DR, Shuler ML (2016). Modular, pumpless body-on-a-chip platform for the co-culture of GI tract epithelium and 3D primary liver tissue. Lab Chip.

[7] Fan YQ (2018). Applications of modular microfluidics technology. Chin J Anal Chem.

[8] Lai XC, Yang MP, Wu H, Li DC (2022). Modular microfluidics: current status and future prospects. Micromachines.

[9] Bhuiyan NH, Hong JH, Uddin MJ, Shim JS (2022). Artificial intelligence-controlled microfluidic device for fluid automation and bubble removal of immunoassay operated by a smartphone. Anal Chem.

[10] Nafea M, Nawabjan A, Ali MSM (2018). A wirelessly-controlled piezoelectric microvalve for regulated drug delivery. Sens Actuat Phys.

[11] Qian JY, Hou CW, Li XJ, Jin ZJ (2020). Actuation mechanism of microvalves: a review. Micromachines.

[12] Oh KW, Ahn CH (2006). A review of microvalves. J Micromech Microeng.

[13] T. Aravind, S.P. Kumar, G. Raj, P. Prasanth, P.S. Gobinath. A novel thermopneumatic based micropump and microvalve using phase change liquid. 2013 IEEE International Conference on, IEEE (2013), pp. 66-69, 10.1109/ICSSS.2013.6623002. https://ieeexplore.ieee.org/document/6623002.

[14] Bae B, Han J, Masel RI, Shannon MA (2007). A bidirectional electrostatic microvalve with microsecond switching performance. J Microelectromech Syst.

[15] Kim H, Astle AA, Najafi K, Bernal LP, Washabaugh PD (2015). An integrated electrostatic peristaltic 18-stage gas micropump with active microvalves. J Microelectromech Syst.

[16] Sandoughsaz A, Besharatian A, Bernal L P and Najafi K. Modular stacked variable-compression ratio multi-stage gas micropump. In 18th international conference on solid-state sensors, actuators and microsystems (TRANSDUCERS). 2015;704–7. 10.1109/TRANSDUCERS.2015.7181020. https://ieeexplore.ieee.org/abstract/document/7181020.

[17] Pekas N, Zhang Q, Juncker D (2012). Electrostatic actuator with liquid metal-elastomer compliant electrodes used for on-chip microvalving. J Micromechan Microeng.

[18] Gunda A, Ozkayar G, Tichem M, Ghatkesar AMK (2020). Proportional microvalve using a unimorph piezoelectric microactuator. Micromachines.

[19] Zhang DY, Lv JL, Jiang YG, Chen HW, Fu JC (2014). A piezoelectric microvalve with a flexure-hinged driving frame and microfabricated silicon sealing pair. Mechatronics.

[20] Bussmann AB, Durasiewicz CP, Kibler SHA, Wald CK (2021). Piezoelectric titanium based microfluidic pump and valves for implantable medical applications. Sens Actuat Phys.

[21] Groen MS (2014). A piezoelectric micro control valve with integrated capacitive sensing for ambulant blood pressure waveform monitoring. J Micromechan Microeng.

[22] Bintoro JS, Hesketh PJ (2005). An electromagnetic actuated on/off microvalve fabricated on top of a single wafer. J Micromech Microeng.

[23] Beebe DJ (2000). Functional hydrogel structures for autonomous flow control inside microfluidic channels. Nature.

[24] Baldi A, Lei M, Gu YD, Siegel RA, Ziaie B (2006). A microstructured silicon membrane with entrapped hydrogels for environmentally sensitive fluid gating. Sens Actuat Chem.

[25] Lin S, Wang W, Ju XJ, Xie R, Chu LY (2014). A simple strategy for in situ fabrication of a smart hydrogel microvalve within microchannels for thermostatic control. Lab Chip.

[26] D'Eramo L (2018). Microfluidic actuators based on temperature-responsive hydrogels. Microsyst Nanoeng..

[27] Lin XS (2021). NIR-responsive metal-containing polymer hydrogel for light-controlled microvalve. Polym Chem.

[28] Baldi A, Gu YD, Loftness PE, Siegel RA, Ziaie B (2003). A hydrogel-actuated environmentally sensitive microvalve for active flow control. J Microelectromech Syst.

[29] Chang YJ, Chen SC, Hsu CL (2013). Study on microchannel design and burst frequency detection for centrifugal microfluidic system. Adv Mat Sci Eng.

[30] Yamada M, Seki M (2004). Nanoliter-sized liquid dispenser array for multiple biochemical analysis in microfluidic devices. Anal Chem.

[31] Yang BZ, Lin Q (2007). A planar compliance-based self-adaptive microfluid variable resistor. J Microelectromech Syst.

[32] Chang HJ, Ye WB, Kartalov EP (2012). Quantitative modeling of the behaviour of microfluidic autoregulatory devices. Lab Chip.

[33] Hu M (2004). A silicon-on-insulator based micro check valve. J Micromech Microeng.

[34] Chen PJ, Rodger DC, Meng EM, Humayun MS, Tai YC (2007). Surface-micromachined parylene dual valves for on-chip unpowered microflow regulation. J Microelectromech Syst.

[35] Adams ML, Johnston ML, Scherer A, Quake SR (2005). Polydimethylsiloxane based microfluidic diode. J Micromech Microeng.

[36] Perdigones F, Garcia JM, Quero JM (2014). Fabrication process for integration of SU-8 check valves on printed circuit board- based microfluidic platforms. Micro Nano Lett.

[37] Araci IE, Quake SR (2012). Microfluidic very large scale integration (mVLSI) with integrated micromechanical valves. Lab Chip.

[38] Ishida T, McLaughlin D, Tanaka Y, Omata T (2018). First-come-first-store microfluidic device of droplets using hydrophobic passive microvalves. Sens Actuat Chem.

[39] Laser DJ, Santiago JG (2004). A review of micropumps. J Micromech Microeng.

[40] Nguyen NT, Huang XY, Chuan TK (2002). MEMS-micropumps: a review. J Fluids Eng Trans Asme.

[41] Mohith S, Karanth PN, Kulkarni SM (2019). Recent trends in mechanical micropumps and their applications: a review. Mechatronics.

[42] Wang YN, Fu LM (2018). Micropumps and biomedical applications—a review. Microelectron Eng.

[43] Zeng Y, Shin MM, Wang TY (2013). Programmable active droplet generation enabled by integrated pneumatic micropumps. Lab Chip.

[44] Chia BT, Liao HH, Yang YJ (2011). A novel thermo-pneumatic peristaltic micropump with low temperature elevation on working fluid. Sens Actuat Phys.

[45] Chee PS, Minjal MN, Leow PL, Ali MSM (2015). Wireless powered thermo-pneumatic micropump using frequency-controlled heater. Sens Actuat Phys.

[46] Saif MTA, Alaca BE, Sehitoglu H (1999). Analytical modeling of electrostatic membrane actuator for micro pumps. J Microelectromech Syst.

[47] Wang XY, Ma YT, Yan GY, Feng ZH (2014). A compact and high flow-rate piezoelectric micropump with a folded vibrator. Smart Mat Struct.

[48] Rusli MQA, Chee PS, Arsat R, Lau KX, Leow PL (2018). Electromagnetic actuation dual-chamber bidirectional flow micropump. Sens Actuat Phys.

[49] Mi SL, Pu HT, Xia SY, Sun W (2020). A minimized valveless electromagnetic micropump for microfluidic actuation on organ chips. Sens Actuat Phys.

[50] Ahn CH, Allen MG. In Proceedings IEEE micro electro mechanical systems. 1995. 408 (IEEE).

[51] Dopper J (1997). Micro gear pumps for dosing of viscous fluids. J Micromech Microeng.

[52] Hainan C, Zhaoying Z, Yong L, Xiongying Y, Yihua Y. in 1997 International symposium on micromechanics and human science (Cat. No. 97TH8311). 115–117.

[53] Kazemi PZ, Selvaganapathy PR, Ching CY (2009). Effect of electrode asymmetry on performance of electrohydrodynamic micropumps. J Microelectromech Syst.

[54] Kazemi PZ, Selvaganapathy PR, Ching CY (2009). Electrohydrodynamic micropumps with asymmetric electrode geometries for microscale electronics cooling. IEEE Trans Dielectr Electr Insul.

[55] Singhal V, Garimella SV (2005). A novel valveless micropump with electrohydrodynamic enhancement for high heat flux cooling. IEEE Trans Adv Packag.

[56] Hossan MR, Dutta D, Islam N, Dutta P (2018). Review: electric field driven pumping in microfluidic device. Electrophoresis.

[57] Seyed-Yagoobi, J. Electrohydrodynamic induction pumping of dielectric liquid films. 15th IEEE International conference on dielectric liquids. 2005;155–160. 10.1109/ICDL.2005.1490050. https://ieeexplore.ieee.org/abstract/document/1490050.

[58] Lemoff AV, Lee AP (2000). An AC magnetohydrodynamic micropump. Sens Act Chem.

[59] Hasan MI, Ali AJF, Tufah RS (2017). Numerical study of the effect of channel geometry on the performance of magnetohydrodynamic micro pump. Eng Sci Technol Int J.

[60] Zhao GP, Jian YJ, Chang L, Buren M (2015). Magnetohydrodynamic flow of generalized Maxwell fluids in a rectangular micropump under an AC electric field. J Magn Magn Mater.

[61] Chatterjee D, Amiroudine S (2011). Lattice boltzmann simulation of thermofluidic transport phenomena in a DC magnetohydrodynamic (MHD) micropump. Biomed Microdevice.

[62] Squires TM, Quake SR (2005). Microfluidics: fluid physics at the nanoliter scale. Rev Mod Phys.

[63] Nguyen NT, Wu ZG (2005). Micromixers—a review. J Micromech Microeng.

[64] Andrew Evan Kamholz, Bernhard H. Weigl, Bruce A. Finlayson, and Paul Yager. Quantitative analysis of molecular interaction in a microfluidic channel the T-sensor. Analytical Chemistry. 1999;71:5340-47. 10.1021/ac990504j. 10.1021/ac990504j 10596213

[65] Gobby D, Angeli P, Gavriilidis A (2001). Mixing characteristics of T-type microfluidic mixers. J Micromech Microeng.

[66] Soleymani A, Kolehmainen E, Turunen I (2008). Numerical and experimental investigations of liquid mixing in T-type micromixers. Chem Eng J.

[67] Ismagilov RF, Stroock AD, Kenis PJA, Whitesides G, Stone HA (2000). Experimental and theoretical scaling laws for transverse diffusive broadening in two-phase laminar flows in microchannels. Appl Phys Lett.

[68] Lee CY, Wang WT, Liu CC, Fu LM (2016). Passive mixers in microfluidic systems: a review. Chem Eng J.

[69] Schonfeld F, Hessel V, Hofmann C (2004). An optimised split-and-recombine micro-mixer with uniform ‘chaotic’ mixing. Lab Chip.

[70] Lin Y, Gerfen GJ, Rousseau DL, Yeh SR (2003). Ultrafast microfluidic mixer and freeze-quenching device. Anal Chem.

[71] Zhou BP (2015). Design and fabrication of magnetically functionalized flexible micropillar arrays for rapid and controllable microfluidic mixing. Lab Chip.

[72] Hong CC, Choi JW, Ahn CH (2004). A novel in-plane passive microfluidic mixer with modified tesla structures. Lab Chip.

[73] Hossain S, Ansari MA, Husain A, Kim KY (2010). Analysis and optimization of a micromixer with a modified Tesla structure. Chem Eng J.

[74] Liu RH (2000). Passive mixing in a three-dimensional serpentine microchannel. J Microelectromech Syst.

[75] Vijayendran RA, Motsegood KM, Beebe DJ, Leckband DE (2003). Evaluation of a three-dimensional micromixer in a surface-based biosensor. Langmuir.

[76] Stroock AD (2002). Chaotic mixer for microchannels. Science.

[77] Song H, Tice JD, Ismagilov RF (2003). A microfluidic system for controlling reaction networks in time. Angewandte Chem Int Ed.

[78] Tung KY, Li CC, Yang JT (2009). Mixing and hydrodynamic analysis of a droplet in a planar serpentine micromixer. Microfluid Nanofluid.

[79] El Moctar AO, Aubry N, Batton J (2006). Electro-hydrodynamic micro-fluidic mixer. Houille Blanche.

[80] Modarres P, Tabrinan M (2020). Electrohydrodynamic-driven micromixing for the synthesis of highly monodisperse nanoscale liposomes. Acs Appl Nano Mater.

[81] Oddy MH, Santiago JG, Mikkelsen JC (2001). Electrokinetic instability micromixing. Anal Chem.

[82] Modarres P, Tabrizian M (2020). Phase-controlled field-effect micromixing using AC electroosmosis. Microsyst Nanoeng..

[83] Bau HH, Zhong JH, Yi MQ (2001). A minute magneto hydro dynamic (MHD) mixer. Sens Actuat Chem.

[84] Wang Y, Zhe J, Chung BTF, Dutta P (2008). A rapid magnetic particle driven micromixer. Microfluid Nanofluid.

[85] Chen XY, Zhang L (2017). A review on micromixers actuated with magnetic nanomaterials. Microchim Acta.

[86] Yaralioglu GG, Wygant IO, Marentis TC, Khuri-Yakub BT (2004). Ultrasonic mixing in microfluidic channels using integrated transducers. Anal Chem.

[87] Thurgood P (2023). Dynamic vortex generation, pulsed injection, and rapid mixing of blood samples in microfluidics using the tube oscillation mechanism. Anal Chem.

[88] Liu RH, Yang JN, Pindera MZ, Athavale M, Grodzinski P (2002). Bubble-induced acoustic micromixing. Lab Chip.

[89] Liu RH, Lenigk R, Druyor-Sanchez RL, Yang JN, Grodzinski P (2003). Hybridization enhancement using cavitation microstreaming. Anal Chem.

[90] Deshmukh, AA, Liepmann, D, Pisano, AP. Continuous micromixer with pulsatile micropumps. In Technical digest of the IEEE solid state sensor and actuator workshop (Hilton Head Island, SC). https://citeseerx.ist.psu.edu/document?repid=rep1&type=pdf&doi=ce5fc45ff0174c09a8275e292bc8669c25717c8b.

[91] Glasgow I, Aubry N (2003). Enhancement of microfluidic mixing using time pulsing. Lab Chip.

[92] Niu XZ, Lee YK (2003). Efficient spatial-temporal chaotic mixing in microchannels. J Micromech Microeng.

[93] Sajeesh P, Sen AK (2014). Particle separation and sorting in microfluidic devices: a review. Microfluid Nanofluid.

[94] Pohl HA (1951). The motion and precipitation of suspensoids in divergent electric fields. J Appl Phys.

[95] Zhang J (2018). Tunable particle separation in a hybrid dielectrophoresis (DEP)-inertial microfluidic device. Sens Actuat Chem.

[96] Choi S, Park JK (2005). Microfluidic system for dielectrophoretic separation based on a trapezoidal electrode array. Lab Chip.

[97] Barrett LM, Skulan AJ, Singh AK, Cummings EB, Fiechtner GJ (2005). Dielectrophoretic manipulation of particles and cells using insulating ridges in faceted prism microchannels. Anal Chem.

[98] Luo JH, Muratore KA, Arriaga EA, Ros A (2016). Deterministic absolute negative mobility for micro- and submicrometer particles induced in a microfluidic device. Anal Chem.

[99] Petersson F, Nilsson A, Holm C, Jonsson H, Laurell T (2004). Separation of lipids from blood utilizing ultrasonic standing waves in microfluidic channels. Analyst.

[100] Petersson F, Nilsson A, Holm C, Jonsson H, Laurell T (2005). Continuous separation of lipid particles from erythrocytes by means of laminar flow and acoustic standing wave forces. Lab Chip.

[101] Ashkin A, Dziedzic JM, Bjorkholm JE, Chu S (1986). Observation of a single-beam gradient force optical trap for dielectric particles. Opt Lett.

[102] Ashkin A (1997). Optical trapping and manipulation of neutral particles using lasers. Proc Natl Acad Sci USA.

[103] MacDonald MP, Spalding GC, Dholakia K (2003). Microfluidic sorting in an optical lattice. Nature.

[104] Miltenyi S, Müller W, Weichel W, Radbruch A (1990). High gradient magnetic cell separation with MACS. Cytom J Int Soc Anal Cytol.

[105] Hejazian M, Li WH, Nguyen NT (2015). Lab on a chip for continuous-flow magnetic cell separation. Lab Chip.

[106] Pamme N (2006). Magnetism and microfluidics. Lab Chip.

[107] Zhu TT, Marrero F, Mao LD (2010). Continuous separation of non-magnetic particles inside ferrofluids. Microfluid Nanofluid.

[108] Yamada M, Nakashima M, Seki M (2004). Pinched flow fractionation: continuous size separation of particles utilizing a laminar flow profile in a pinched microchannel. Anal Chem.

[109] Huang LR, Cox EC, Austin RH, Sturm JC (2004). Continuous particle separation through deterministic lateral displacement. Science.

[110] Inglis DW, Davis JA, Austin RH, Sturm JC (2006). Critical particle size for fractionation by deterministic lateral displacement. Lab Chip.

[111] Yamada M, Seki M (2005). Hydrodynamic filtration for on-chip particle concentration and classification utilizing microfluidics. Lab Chip.

[112] Yamada M, Seki M (2006). Microfluidic particle sorter employing flow splitting and recombining. Anal Chem.

[113] Segre G, Silberberg A (1961). Radial particle displacements in poiseuille flow of suspensions. Nature.

[114] Park JS, Jung HI (2009). Multiorifice flow fractionation: continuous size-based separation of microspheres using a series of contraction/expansion microchannels. Anal Chem.

[115] Bhagat AAS, Kuntaegowdanahalli SS, Papautsky I (2008). Continuous particle separation in spiral microchannels using dean flows and differential migration. Lab Chip.

[116] Yuan D (2019). Sheathless separation of microalgae from bacteria using a simple straight channel based on viscoelastic microfluidics. Lab Chip.

[117] Zhang J (2016). Fundamentals and applications of inertial microfluidics: a review. Lab Chip.

[118] Griffiths AD, Tawfik DS (2006). Miniaturising the laboratory in emulsion droplets. Trends Biotechnol.

[119] Stone HA, Stroock AD, Ajdari A (2004). Engineering flows in small devices: microfluidics toward a lab-on-a-chip. Annu Rev Fluid Mech.

[120] Dubay R, Urban JN, Darling EM (2021). Single-cell microgels for diagnostics and therapeutics. Adv Functional Mater.

[121] Shang LR, Cheng Y, Zhao YJ (2017). Emerging droplet microfluidics. Chem Rev.

[122] Christopher GF, Anna SL (2007). Microfluidic methods for generating continuous droplet streams. J Phys Appl Phys.

[123] Umbanhowar PB, Prasad V, Weitz DA (2000). Monodisperse emulsion generation via drop break off in a coflowing stream. Langmuir.

[124] Thorsen T, Roberts RW, Arnold FH, Quake SR (2001). Dynamic pattern formation in a vesicle-generating microfluidic device. Phys Rev Lett.

[125] Nisisako T, Torii T, Higuchi T (2002). Droplet formation in a microchannel network. Lab Chip.

[126] Xu JH, Li SW, Tan J, Wang YJ, Luo GS (2006). Preparation of highly monodisperse droplet in a T-junction microfluidic device. AIChE J.

[127] Anna SL, Bontoux N, Stone HA (2003). Formation of dispersions using “flow focusing” in microchannels. Appl Phys Lett.

[128] Link DR (2006). Electric control of droplets in microfluidic devices. Angewandte Chem Int Ed.

[129] Tan SH, Semin B, Baret JC (2014). Microfluidic flow-focusing in ac electric fields. Lab Chip.

[130] Tan SH, Nguyen NT, Yobas L, Kang TG (2010). Formation and manipulation of ferrofluid droplets at a microfluidic T-junction. J Micromechan Microeng.

[131] Tan SH, Nguyen NT (2011). Generation and manipulation of monodispersed ferrofluid emulsions: the effect of a uniform magnetic field in flow-focusing and T-junction configurations. Phys Rev.

[132] Schmid L, Franke T (2013). SAW-controlled drop size for flow focusing. Lab Chip.

[133] Collins DJ, Alan T, Helmerson K, Neild A (2013). Surface acoustic waves for on-demand production of picoliter droplets and particle encapsulation. Lab Chip.

[134] Thurgood P (2021). Generation of programmable dynamic flow patterns in microfluidics using audio signals. Lab Chip.

[135] Nguyen NT (2007). Thermally mediated droplet formation in microchannels. Appl Phys Lett.

[136] Baroud CN, Delville JP, Gallaire F, Wunenburger R (2007). Thermocapillary valve for droplet production and sorting. Phys Rev.

[137] Zhu PA, Wang LQ (2017). Passive and active droplet generation with microfluidics: a review. Lab Chip.

[138] Chen CT, Lee GB (2006). Formation of microdroplets in liquids utilizing active pneumatic choppers on a microfluidic chip. J Microelectromech Syst.

[139] Willaime H, Barbier V, Kloul L, Maine S, Tabeling P (2006). Arnold tongues in a microfluidic drop emitter. Phys Rev Lett.

[140] Yin ZQ, Huang ZM, Lin XH, Gao XY, Bao FB (2020). Droplet generation in a flow-focusing microfluidic device with external mechanical vibration. Micromachines.

[141] Keenan TM, Folch A (2008). Biomolecular gradients in cell culture systems. Lab Chip.

[142] Lin F (2004). Generation of dynamic temporal and spatial concentration gradients using microfluidic devices. Lab Chip.

[143] Haessler U, Pisano M, Wu MM, Swartz MA (2011). Dendritic cell chemotaxis in 3D under defined chemokine gradients reveals differential response to ligands CCL21 and CCL19. Proc Natl Acad Sci USA.

[144] Sun K, Wang ZX, Jiang XY (2008). Modular microfluidics for gradient generation. Lab Chip.

[145] Wang X, Liu ZM, Pang Y (2017). Concentration gradient generation methods based on microfluidic systems. RSC Adv.

[146] Jeon NL (2000). Generation of solution and surface gradients using microfluidic systems. Langmuir.

[147] Oh KW, Lee K, Ahn B, Furlani EP (2012). Design of pressure-driven microfluidic networks using electric circuit analogy. Lab Chip.

[148] Holden MA, Kumar S, Castellana ET, Beskok A, Cremer PS (2003). Generating fixed concentration arrays in a microfluidic device. Sens Actuat Chem.

[149] Zhou Y, Wang Y, Mukherjee T, Lin Q (2009). Generation of complex concentration profiles by partial diffusive mixing in multi-stream laminar flow. Lab Chip.

[150] Abhyankar VV, Lokuta MA, Huttenlocher A, Beebe DJ (2006). Characterization of a membrane-based gradient generator for use in cell-signaling studies. Lab Chip.

[151] Abhyankar VV (2008). A platform for assessing chemotactic migration within a spatiotemporally defined 3D microenvironment. Lab Chip.

[152] Cheng SY (2007). A hydrogel-based microfluidic device for the studies of directed cell migration. Lab Chip.

[153] Song H, Ismagilov RF (2003). Millisecond kinetics on a microfluidic chip using nanoliters of reagents. J Am Chem Soc.

[154] Bui MPN (2011). Enzyme kinetic measurements using a droplet-based microfluidic system with a concentration gradient. Anal Chem.

[155] Cai LF, Zhu Y, Du GS, Fang Q (2012). Droplet-based microfluidic flow injection system with large-scale concentration gradient by a single nanoliter-scale injection for enzyme inhibition assay. Anal Chem.

[156] Chao TC, Ros A (2008). Microfluidic single-cell analysis of intracellular compounds. J R Soc Interface.

[157] Luan QY, Macaraniag C, Zhou J, Papautsky I (2020). Microfluidic systems for hydrodynamic trapping of cells and clusters. Biomicrofluidics.

[158] Johann RM (2006). Cell trapping in microfluidic chips. Anal Bioanal Chem.

[159] Deng YL, Guo Y, Xu B (2020). Recent development of microfluidic technology for cell trapping in single cell analysis: a review. Processes.

[160] Nilsson J, Evander M, Hammarstrom B, Laurell T (2009). Review of cell and particle trapping in microfluidic systems. Anal Chim Acta.

[161] Liu XY (2021). Integrating a concentration gradient generator and a single-cell trapper array for high-throughput screening the bioeffects of nanomaterials. Angewandte Chem Int Ed.

[162] Sarioglu AF (2015). A microfluidic device for label-free, physical capture of circulating tumor cell clusters. Nat Methods.

[163] Rettig JR, Folch A (2005). Large-scale single-cell trapping and imaging using microwell arrays. Anal Chem.

[164] Fallahi H (2022). On-demand deterministic release of particles and cells using stretchable microfluidics. Nanoscale Horizons.

[165] Wang RJ (2013). Hydrodynamic trapping of particles in an expansion-contraction microfluidic device. Abstr Appl Anal.

[166] Yeo T (2016). Microfluidic enrichment for the single cell analysis of circulating tumor cells. Sci Rep.

[167] Arakawa T, Noguchi M, Sumitomo K, Yamaguchi Y, Shoji S (2011). High-throughput single-cell manipulation system for a large number of target cells. Biomicrofluidics.

[168] Tan WH, Takeuchi S (2007). A trap-and-release integrated microfluidic system for dynamic microarray applications. Proc Natl Acad Sci USA.

[169] Zhou Y (2016). A microfluidic platform for trapping, releasing and super-resolution imaging of single cells. Sens Actuat Chem.

[170] Chen C (2020). Dynamic screening and printing of single cells using a microfluidic chip with dual microvalves. Lab Chip.

[171] Shemesh J (2014). Stationary nanoliter droplet array with a substrate of choice for single adherent/nonadherent cell incubation and analysis. Proc Natl Acad Sci USA.

[172] Hassanzadeh-Barforoushi A (2018). Static droplet array for culturing single live adherent cells in an isolated chemical microenvironment. Lab Chip.

[173] Hassanzadeh-Barforoushi A, Warkiani ME, Gallego-Ortega D, Liu GZ, Barber T (2020). Capillary-assisted microfluidic biosensing platform captures single cell secretion dynamics in nanoliter compartments. Biosens Bioelect.

[174] Shake T (2013). Embedded passivated-electrode insulator-based dielectrophoresis (E pi DEP). Anal Bioanal Chem.

[175] Nguyen NV, Jen CP (2018). Impedance detection integrated with dielectrophoresis enrichment platform for lung circulating tumor cells in a microfluidic channel. Biosens Bioelectron.

[176] Ramadan Q, Samper V, Poenar DP, Yu C (2006). An integrated microfluidic platform for magnetic microbeads separation and confinement. Biosens Bioelectron.

[177] Sun XC (2017). An integrated microfluidic system using a micro-fluxgate and micro spiral coil for magnetic microbeads trapping and detecting. Sci Rep.

[178] Furlani EP, Sahoo Y, Ng KC, Wortman JC, Monk TE (2007). A model for predicting magnetic particle capture in a microfluidic bioseparator. Biomed Microdevice.

[179] Liu SJ (2019). Miniaturized optical fiber tweezers for cell separation by optical force. Opt Lett.

[180] Liu XS, Huang JB, Li YC, Zhang Y, Li BJ (2017). Optofluidic organization and transport of cell chain. J Biophotonics.

[181] Collins DJ, Alan T, Neild A (2014). The particle valve: On-demand particle trapping, filtering, and release from a microfabricated polydimethylsiloxane membrane using surface acoustic waves. Appl Phys Lett.

[182] Broman A (2021). Multinodal acoustic trapping enables high capacity and high throughput enrichment of extracellular vesicles and microparticles in miRNA and MS proteomics studies. Anal Chem.

[183] Gupta N (2016). Microfluidics-based 3D cell culture models: utility in novel drug discovery and delivery research. Bioeng Transl Med.

[184] van Duinen V, Trietsch SJ, Joore J, Vulto P, Hankemeier T (2015). Microfluidic 3D cell culture: from tools to tissue models. Curr Opin Biotechnol.

[185] Liu RR (2022). From 2D to 3D co-culture systems: a review of co-culture models to study the neural cells interaction. Internat J Mol Sci.

[186] Shang ML, Soon RH, Lim CT, Khoo BL, Han J (2019). Microfluidic modelling of the tumor microenvironment for anti-cancer drug development. Lab Chip.

[187] Gu W, Zhu XY, Futai N, Cho BS, Takayama S (2004). Computerized microfluidic cell culture using elastomeric channels and Braille displays. Proc Natl Acad Sci USA.

[188] Paschos NK, Brown WE, Eswaramoorthy R, Hu JC, Athanasiou KA (2015). Advances in tissue engineering through stem cell-based co-culture. J Tissue Eng Regen Med.

[189] Burmeister A, Grunberger A (2020). Microfluidic cultivation and analysis tools for interaction studies of microbial co-cultures. Curr Opin Biotechnol.

[190] Huh D (2007). Acoustically detectable cellular-level lung injury induced by fluid mechanical stresses in microfluidic airway systems. Proc Natl Acad Sci USA.

[191] Song JW (2009). Microfluidic endothelium for studying the intravascular adhesion of metastatic breast cancer cells. Plos ONE.

[192] Young EWK, Beebe DJ (2010). Fundamentals of microfluidic cell culture in controlled microenvironments. Chem Soc Rev.

[193] Kim L, Vahey MD, Lee HY, Voldman J (2006). Microfluidic arrays for logarithmically perfused embryonic stem cell culture. Lab Chip.

[194] Lee KG (2014). 3D printed modules for integrated microfluidic devices. RSC Adv.

[195] Zhou ZY, Kong TT, Mkaouar H, Salama KN, Zhang JM (2018). A hybrid modular microfluidic device for emulsion generation. Sens Actuat Phys.

[196] Warkiani ME (2014). An ultra-high-throughput spiral microfluidic biochip for the enrichment of circulating tumor cells. Analyst.

[197] Rhee M, Burns MA (2008). Microfluidic assembly blocks. Lab Chip.

[198] Gray BL, Collins SD, Smith RL (2004). Interlocking mechanical and fluidic interconnections for microfluidic circuit boards. Sens Actuat Phys.

[199] Langelier SM (2011). Flexible casting of modular self-aligning microfluidic assembly blocks. Lab Chip.

[200] Owens CE, Hart AJ (2018). High-precision modular microfluidics by micromilling of interlocking injection-molded blocks. Lab Chip.

[201] Xie X, Maharjan S, Liu SW, Zhang YS, Livermore C (2020). A Modular, reconfigurable microfabricated assembly platform for microfluidic transport and multitype cell culture and drug testing. Micromachines.

[202] Qiu JJ, Gao Q, Zhao HM, Fu JZ, He Y (2017). Rapid customization of 3D integrated microfluidic chips via modular structure-based design. ACS Biomater Sci Eng.

[203] Nie J (2018). 3D printed Lego (R)-like modular microfluidic devices based on capillary driving. Biofabrication.

[204] Loskill P, Marcus SG, Mathur A, Reese WM, Healy KE (2015). mu organo: a lego (R)-like plug & play system for modular multi-organ-chips. Plos ONE.

[205] Atencia J (2010). Magnetic connectors for microfluidic applications. Lab Chip.

[206] Wagler PF, Tangen U, Ott J, McCaskill JS (2015). General-purpose, parallel and reversible microfluidic interconnects. Ieee Trans Comp Packaging Manufact Technol.

[207] Pfreundt A, Andersen KB, Dimaki M, Svendsen WE (2015). An easy-to-use microfluidic interconnection system to create quick and reversibly interfaced simple microfluidic devices. J Micromech Microeng.

[208] Li CY, Wang XX, Xu J, Ma B (2020). One-step liquid molding based modular microfluidic circuits. Analyst.

[209] Cha HT (2022). Tuning particle inertial separation in sinusoidal channels by embedding periodic obstacle microstructures. Lab Chip.

[210] Lee YS, Lu YT, Chang CM, Liu CH (2022). Finger-powered cell-sorting microsystem chip for cancer-study applications. Sens Actuat Chem.

[211] Millet LJ, Lucheon JD, Standaert RF, Retterer ST, Doktycz MJ (2015). Modular microfluidics for point-of-care protein purifications. Lab Chip.

[212] Okura N (2017). A compact and facile microfluidic droplet creation device using a piezoelectric diaphragm micropump for droplet digital PCR platforms. Electrophoresis.

[213] Zhang YS (2017). Multisensor-integrated organs-on-chips platform for automated and continual in situ monitoring of organoid behaviors. Proc Natl Acad Sci USA.

[214] Panariello L (2020). A modular millifluidic platform for the synthesis of iron oxide nanoparticles with control over dissolved gas and flow configuration. Materials.

[215] Perozziello G (2014). Microfluidics & nanotechnology: towards fully integrated analytical devices for the detection of cancer biomarkers. RSC Adv.

[216] Bovard D (2018). A lung/liver-on-a-chip platform for acute and chronic toxicity studies. Lab Chip.

[217] Hlawatsch, N. et al. A lab-on-a-chip system for the development of complex assays using modular microfluidic components. In Conference on Microfluidics, BioMEMS, and Medical Microsystems X. 2012. 10.1117/12.910269.

[218] Skardal A (2017). Multi-tissue interactions in an integrated three-tissue organ-on-a-chip platform. Sci Rep.

[219] Kampe T, Konig A, Schroeder H, Hengstler JG, Niemeyer CM (2014). Modular microfluidic system for emulation of human phase I/phase II Metabolism. Anal Chem.

[220] Munshi AS, Chen CP, Townsend AD, Martin RS (2018). Use of 3D printing and modular microfluidics to integrate cell culture, injections and electrochemical analysis. Anal Methods.

[221] Vasilescu SA, Bazaz SR, Jin DY, Shimoni O, Warkiani ME (2020). 3D printing enables the rapid prototyping of modular microfluidic devices for particle conjugation. Appl Mater Today.

[222] Bandulasena MV, Vladisavljevic GT, Benyahia B (2019). Versatile reconfigurable glass capillary microfluidic devices with Lego (R) inspired blocks for drop generation and micromixing. J Colloid Interface Sci.

[223] Liou DS, Hsieh YF, Kuo LS, Yang CT, Chen PH (2011). Modular component design for portable microfluidic devices. Microfluid Nanofluid.

[224] Liu B (2022). High-throughput microfluidic production of bimetallic nanoparticles on mxene nanosheets and application in hydrogen peroxide detection. Acs Appl Mater Interfaces.

[225] Yuen PK, Bliss JT, Thompson CC, Peterson RC (2009). Multidimensional modular microfluidic system. Lab Chip.

[226] Bhargava KC, Thompson B, Malmstadt N (2014). Discrete elements for 3D microfluidics. Proc Natl Acad Sci USA.

[227] Hsieh YF (2014). A Lego (R)-like swappable fluidic module for bio-chem applications. Sens Actuat Chem.

[228] Tsuda S (2015). Customizable 3D printed ‘plug and play’ millifluidic devices for programmable fluidics. Plos ONE.

[229] Chen XJ, Mo DY, Gong MF (2020). 3D printed reconfigurable modular microfluidic system for generating gel microspheres. Micromachines.

[230] Zhang YX (2020). Modular off-chip emulsion generator enabled by a revolving needle. Lab Chip.

[231] Lai XC (2020). A Rubik's microfluidic cube. Microsys Nanoeng..

[232] Perozziello G, Bundgaard F, Geschke O (2008). Fluidic interconnections for microfluidic systems: a new integrated fluidic interconnection allowing plug ‘n’ play functionality. Sens Actuat Chem.

[233] Godino N (2010). Construction and characterisation of a modular microfluidic system: coupling magnetic capture and electrochemical detection. Microfluid Nanofluid.

[234] Warkiani ME (2016). Ultra-fast, label-free isolation of circulating tumor cells from blood using spiral microfluidics. Nat Protoc.

[235] Ren H (2021). Multiplexed serpentine microchannels for high-throughput sorting of disseminated tumor cells from malignant pleural effusion. Sens Actuat Chem.

[236] Xu X (2022). 3D-stacked multistage inertial microfluidic chip for high-throughput enrichment of circulating tumor cells. Cyborg Bionic Sys.

[237] Jiang FT, Xiang N (2021). Series and parallel integration of flow regulators for precise and multiple-output fluid delivery. Sens Actuat Phys.

[238] Zhang XJ (2016). Passive flow regulator for precise high-throughput flow rate control in microfluidic environments. RSC Adv.

[239] Guo KF (2022). A novel 3D tesla valve micromixer for efficient mixing and chitosan nanoparticle production. Electrophoresis.

[240] Jiang FT, Xiang N (2022). Integrated microfluidic handheld cell sorter for high-throughput label-free malignant tumor cell sorting. Anal Chem.

[241] Warkiani ME, Tay AKP, Guan GF, Han J (2015). Membrane-less microfiltration using inertial microfluidics. Sci Rep.

[242] Deng NN (2011). Simple and cheap microfluidic devices for the preparation of monodisperse emulsions. Lab Chip.

[243] Glick CC (2016). Rapid assembly of multilayer microfluidic structures via 3D-printed transfer molding and bonding. Microsys Nanoeng.

[244] Lee TY (2018). Accurate, predictable, repeatable micro-assembly technology for polymer, microfluidic modules. Sens Actuat Chem.

[245] Nguyen PQM (2022). Modular micro-PCR system for the onsite rapid diagnosis of COVID-19. Microsys Nanoeng.

[246] Shaikh KA (2005). A modular microfluidic architecture for integrated biochemical analysis. Proc Natl Acad Sci USA.

[247] Han TT, Zhang L, Xu H, Xuan J (2017). Factory-on-chip: Modularised microfluidic reactors for continuous mass production of functional materials. Chem Eng J.

[248] Huang YC (2018). Design criteria and applications of multi-channel parallel microfluidic module. J Micromech Microeng.

[249] Zhang YX (2021). Modular and self-contained microfluidic analytical platforms enabled by magnetorheological elastomer microactuators. Micromachines.

[250] Hourlier-Fargette A (2020). Skin-interfaced soft microfluidic systems with modular and reusable electronics for in situ capacitive sensing of sweat loss, rate and conductivity. Lab Chip.

[251] Fang YH, Zhu S, Cheng WQ, Ni ZH, Xiang N (2022). Efficient bioparticle extraction using a miniaturized inertial microfluidic centrifuge. Lab Chip.

[252] Kong TF, Peng WK, Luong TD, Nguyen NT, Han J (2012). Adhesive-based liquid metal radio-frequency microcoil for magnetic resonance relaxometry measurement. Lab Chip.

[253] Ong LJY (2019). Self-aligning tetris-like (TILE) modular microfluidic platform for mimicking multi-organ interactions. Lab Chip.

[254] Zhu JY (2019). Reconfigurable, self-sufficient convective heat exchanger for temperature control of microfluidic systems. Anal Chem.

[255] Gupta GP, Massague J (2006). Cancer metastasis: building a framework. Cell.

[256] Esmaeilsabzali H, Beischlag TV, Cox ME, Parameswaran AM, Park EJ (2013). Detection and isolation of circulating tumor cells: principles and methods. Biotechnol Adv.

[257] Cristofanilli M (2004). Circulating tumor cells, disease progression, and survival in metastatic breast cancer. N Engl J Med.

[258] Khot PD, Fredricks DNJE. PCR-based diagnosis of human fungal infections. Expert Review of Anti-infective Therapy. 2009;7:1201–21. 10.1586/eri.09.104. 10.1586/eri.09.104 PMC2845394 19968513

[259] Zhu H (2020). PCR past, present and future. Biotechniques.

[260] Chen PC, Nikitopoulos DE, Soper SA, Murphy MC (2008). Temperature distribution effects on micro-CFPCR performance. Biomed Microdevice.

[261] Hashimoto M, Barany F, Xu F, Soper SA (2007). Serial processing of biological reactions using flow-through microfluidic devices: coupled PCR/LDR for the detection of low-abundant DNA point mutations. Analyst.

[262] Sinville R (2008). Ligase detection reaction for the analysis of point mutations using free-solution conjugate electrophoresis in a polymer microfluidic device. Electrophoresis.

[263] Hansen G (2021). Clinical performance of the point-of-care cobas liat for detection of SARS-CoV-2 in 20 minutes: a multicenter study. J Clin Microbiol.

[264] Zhang BY, Korolj A, Lai BFL, Radisic M (2018). Advances in organ-on-a-chip engineering. Nat Rev Mater.

[265] Zhao QB, Cole T, Zhang YX, Tang SY (2021). Mechanical strain-enabled reconstitution of dynamic environment in organ-on-a-chip platforms: a review. Micromachines.

[266] Wu RQ, Zhao XF, Wang ZY, Zhou M, Chen QM (2011). Novel molecular events in oral carcinogenesis via integrative approaches. J Dent Res.

[267] Anselmo AC, Mitragotri S (2016). Nanoparticles in the clinic. Bioeng Transl Med.

[268] Sun JS, Xianyu YL, Jiang XY (2014). Point-of-care biochemical assays using gold nanoparticle-implemented microfluidics. Chem Soc Rev.

[269] Vlachopoulos A (2022). Poly(Lactic Acid)-based microparticles for drug delivery applications: an overview of recent advances. Pharmaceutics.

[270] Niculescu AG, Chircov C, Birca AC, Grumezescu AM (2021). Fabrication and applications of microfluidic devices: a review. Int J Mol Sci.

[271] Hervault A, Thanh NTK (2014). Magnetic nanoparticle-based therapeutic agents for thermo-chemotherapy treatment of cancer. Nanoscale.

[272] Reyes DR, van Heeren H (2019). Proceedings of the first workshop on standards for microfluidics. J Res Nat Inst Stand Technol.

[273] Reyes DR (2021). Accelerating innovation and commercialization through standardization of microfluidic-based medical devices. Lab Chip.

[274] Isozaki A (2020). AI on a chip. Lab Chip.

[275] Dabbagh SR, Rabbi F, Dogan Z, Yetisen AK, Tasoglu S (2020). Machine learning-enabled multiplexed microfluidic sensors. Biomicrofluidics.

[276] Volk AA, Epps RW, Abolhasani M (2021). Accelerated development of colloidal nanomaterials enabled by modular microfluidic reactors: toward autonomous robotic experimentation. Adv Mater.

[277] Zheng JH, Cole T, Zhang YX, Kim J, Tang SY (2021). Exploiting machine learning for bestowing intelligence to microfluidics. Biosens Bioelect.

[278] Seiler ST (2022). Modular automated microfluidic cell culture platform reduces glycolytic stress in cerebral cortex organoids. Sci Rep.

[279] Palleau E, Reece S, Desai SC, Smith ME, Dickey MD (2013). Self-healing stretchable wires for reconfigurable circuit wiring and 3D microfluidics. Adv Mater.

[280] Taylor DL, Panhuis MIH (2016). Self-healing hydrogels. Adv Mater.

[281] Liang YN (2022). Self-healing, self-adhesive and stable organohydrogel-based stretchable oxygen sensor with high performance at room temperature. Nano Micro Lett.

[282] Liu QH, Nian GD, Yang CH, Qu SX, Suo ZG (2018). Bonding dissimilar polymer networks in various manufacturing processes. Nat Commun.

[283] Valentin TM (2019). 3D printed self-adhesive PEGDA-PAA hydrogels as modular components for soft actuators and microfluidics. Polym Chem.

[284] Fallahi H, Zhang J, Nicholls J, Phan HP, Nguyen NT (2020). Stretchable inertial microfluidic device for tunable particle separation. Anal Chem.

[285] Paratore F, Bacheva V, Bercovici M, Kaigala GV (2022). Reconfigurable microfluidics. Nat Rev Chem.

